# Genome-wide analyses of cassava *Pathogenesis-related* (*PR*) gene families reveal core transcriptome responses to whitefly infestation, salicylic acid and jasmonic acid

**DOI:** 10.1186/s12864-019-6443-1

**Published:** 2020-01-29

**Authors:** Maria L. Irigoyen, Danielle C. Garceau, Adriana Bohorquez-Chaux, Luis Augusto Becerra Lopez-Lavalle, Laura Perez-Fons, Paul D. Fraser, Linda L. Walling

**Affiliations:** 1 Department of Botany and Plant Sciences and Institute of Integrative Genome Biology, University of California, Riverside, CA 92521 USA; 2 International Center of Tropical Agriculture (CIAT), Cali, Colombia; 3 Department of Biological Sciences, Royal Holloway University of London, Egham, UK

**Keywords:** Cassava, Jasmonic acid, Pathogenesis-related, *PR* genes, PR proteins, Salicylic acid, Transcriptome, Whitefly, Stress response, Defense, Hormone, Pest

## Abstract

**Background:**

Whiteflies are a threat to cassava (*Manihot esculenta*), an important staple food in many tropical/subtropical regions. Understanding the molecular mechanisms regulating cassava’s responses against this pest is crucial for developing control strategies. Pathogenesis-related (PR) protein families are an integral part of plant immunity. With the availability of whole genome sequences, the annotation and expression programs of the full complement of *PR* genes in an organism can now be achieved. An understanding of the responses of the entire complement of *PR* genes during biotic stress and to the defense hormones, salicylic acid (SA) and jasmonic acid (JA), is lacking. Here, we analyze the responses of cassava *PR* genes to whiteflies, SA, JA, and other biotic aggressors.

**Results:**

The cassava genome possesses 14 of the 17 plant *PR* families, with a total of 447 *PR* genes. A cassava *PR* gene nomenclature is proposed. Phylogenetic relatedness of cassava PR proteins to each other and to homologs in poplar, rice and Arabidopsis identified cassava-specific *PR* gene family expansions. The temporal programs of *PR* gene expression in response to the whitefly (*Aleurotrachelus socialis*) in four whitefly-susceptible cassava genotypes showed that 167 of the 447 *PR* genes were regulated after whitefly infestation. While the timing of *PR* gene expression varied, over 37% of whitefly-regulated *PR* genes were downregulated in all four genotypes. Notably, whitefly-responsive *PR* genes were largely coordinately regulated by SA and JA. The analysis of cassava *PR* gene expression in response to five other biotic stresses revealed a strong positive correlation between whitefly and *Xanthomonas axonopodis* and *Cassava Brown Streak Virus* responses and negative correlations between whitefly and *Cassava Mosaic Virus* responses*.* Finally, certain associations between *PR* genes in cassava expansions and response to biotic stresses were observed among *PR* families.

**Conclusions:**

This study represents the first genome-wide characterization of *PR* genes in cassava. *PR* gene responses to six biotic stresses and to SA and JA are demonstrably different to other angiosperms. We propose that our approach could be applied in other species to fully understand *PR* gene regulation by pathogens, pests and the canonical defense hormones SA and JA.

## Background

Cassava (*Manihot esculenta* Crantz) is grown by small shareholder farmers in more than 100 countries in tropical and subtropical areas, with a production close to 300 million tons [1]. It is a tuberous crop consumed by nearly 800 million people worldwide, especially in Africa where it is a staple food for 500 million people. Cassava is well suited for meeting the challenges imposed by climate change [2, 3], as cassava maintains nearly 50% of its photosynthetic rate under drought conditions [4] and is highly tolerant to acidic soils. However, cassava productivity is endangered by a variety of pests and diseases. Among these crop-damaging pests are whiteflies.

*Aleurotrachelus socialis* Bondar is the most damaging whitefly species in northern South America, particularly Colombia [5, 6]. Whiteflies cause direct damage to their hosts by voracious phloem feeding, honeydew production and subsequent sooty mold growth [7]. In addition, whiteflies (*Bemisia tabaci*) are major vectors of the viruses *Cassava Mosaic Virus* and *Cassava Brown Streak Virus,* which devastate cassava in Eastern and Central Africa [8–11]. Collectively, these attacks produce significant cassava yield losses [12–14]. To reduce the impact of whiteflies on cassava, the identification of new resistance mechanisms and the use of novel transgenic strategies to improve cassava varieties has become increasingly important. A deeper understanding of the molecular basis controlling cassava’s response to whitefly infestation will facilitate these strategies.

Plants have evolved a sophisticated immune system to defend themselves from pests and pathogens, as represented by the multilayered ‘zig-zag’ model [15]. In the first layer, plasma membrane-localized receptors (pattern-recognition receptors) recognize microbe- or pathogen-associated molecular patterns (PAMP) inducing PAMP-triggered immunity [16]. Damage-associated molecular patterns derived from the host after attack, as well as herbivory-associated molecular patterns, can also trigger PAMP-triggered immunity [17]. The second layer involves intracellular receptors, belonging mainly to the nucleotide-binding leucine-rich-repeat (NLR) class, which recognize effectors released by the pathogen/pest to activate effector-triggered immunity [18]. One of the outcomes of this initial recognition and the subsequent signaling cascades is the expression of pathogenesis-related (PR) proteins. First reported in *Tobacco Mosaic Virus*-infected tobacco plants in the early 1970’s [19], PR proteins were later identified in many plant species after infection by a broad range of pathogens [20].

*PR* families are well characterized in Arabidopsis, tomato and potato [21] and are composed of closely related homologs. Currently, there are 17 *PR* families encoding a broad spectrum of activities including glucanases, chitinases, peroxidases, thaumatin-like proteins, and proteases. With the advent of plant whole genome sequences, the complexity of *PR* gene families is beginning to emerge [22–25]. To date, few studies have comprehensively examined expression of the entire complement of *PR* genes in response to multiple biotic stresses or defense hormones.

In this study, we defined the cassava *PR* families and propose a *PR* gene nomenclature. Using phylogenetic trees, we determined the evolutionary relatedness of cassava’s PR proteins to each other and to PR proteins from a dicot (poplar, *Populus trichocarpa*) and a monocot (rice, *Oryza sativa*). To understand cassava’s defense response to phloem-feeding whiteflies, we determined the expression of *PR* genes during whitefly (*Aleurotrachelus socialis*) infestation in four whitefly-susceptible cassava genotypes: COL2246 and COL1468, which are grown in South America, 60444 (one of the few cassava accessions amenable gene transformation technologies), and TME3, which is grown in Africa. Since *PR* genes are often used as markers of SA- and JA-defense responses [21], changes in *PR* gene expression after SA and JA treatments were also determined and correlated with whitefly infestation. Lastly, *PR* gene responses to whiteflies were compared to data sets in the literature that documented responses to five other aggressors: the cassava mealybug *Phenacoccus manihoti*; the bacterial blight pathogen *Xanthomonas axonopodis* pv. *manihotis*; the fungus causing cassava anthracnose disease *Colletotrichum gloeosporioides*, *Cassava Mosaic Virus* (CMV), and *Cassava Brown Streak Virus* (CBSV) [26–33]. Together, our integrative analyses defined the core transcriptome response of susceptible cassava to whitefly infestation, and revealed key *PR* gene families (*PR-2, -5, -7 and -9*) in the responses of cassava to whiteflies, SA, JA, and a variety of other biotic stresses.

## Results

### Cassava *PR* family composition is similar to poplar

Using founder PR proteins defined by van Loon et al. [21] as queries, we identified 447 PR proteins (Additional file 1: Table S1). Proteins within each cassava *PR* family were used to construct phylogenetic trees to establish *PR* gene nomenclature (see Methods). Fourteen of the 17 plant *PR* families were identified in cassava. The *PR-15* and *PR-16* (*PR-15/16* henceforth) families were consolidated because searches using PR-15 and PR-16 founder proteins identified the same set of proteins (Table 1).

**Table 1:** *PR* families of cassava, poplar, rice and Arabidopsis

*PR* –gene family^a^	Function	*Manihot esculenta*	*Populus trichocarpa*	*Oryza sativa*	*Arabidopsis thaliana*
*PR-1*	CAP/SCP superfamily (unknown)	18	14	27	23
*PR-2*	β-1,3-glucanases	50	73	55	70
*PR-3*	Chitinases - Class I, II, IV, VI, VII	22	16	17	21
*PR-4*	Endochitinases	5	3	6	6
*PR-5*	Thaumatin-like	36	39	31	42
*PR-6*	Proteinase inhibitors	3	16	4	7
*PR-7*	Aspartic endoproteases	72	70	55	78
*PR-8*	Chitinases - Class III	10	11	26	1
*PR-9*	Lignin-forming peroxidases	110	88	113	97
*PR-10*	Ribonuclease-like	21	26	8	3
*PR-11*	Chitinases - Class V	5	7	2	9
*PR-12*	Defensins	0	0	2	13
*PR-13*	Thionins	0	0	2	4
*PR-14*	Lipid transfer proteins	30	19	20	23
*PR-15/16*	Oxalate oxidase/Germin-like	59	48	42	74
*PR-17*	unknown	6	7	4	8
Total		447	437	414	479

^a^Founder proteins used as query for each family can be found in Additional file 1: Table S1

To ground our knowledge within the context of angiosperm evolution, we identified the PR proteins from poplar (*Populus trichocarpa*), rice (*Oryza sativa*) and *Arabidopsis thaliana* (see Methods) (Table 1). The total number of *PR* genes ranged from 414 in rice to 479 in Arabidopsis. Similar *PR* family composition was observed in cassava and poplar. For example, *PR-12* and *PR-13* families were absent in cassava and poplar but present in Arabidopsis and rice. Additionally, the *PR-10* family was larger in both cassava (21 genes) and poplar (26 genes) relative to rice (8 genes) and Arabidopsis (3 genes) (Table 1).

### Phylogenetic analysis and physical location of cassava *PR* genes

To investigate the evolution of cassava’s *PR* families, we constructed phylogenetic trees for PR proteins of cassava, poplar, rice, and the founder PR protein(s) for each *PR* family [21] (Additional file 2: Figure S1-S14). We observed that for some *PR* families (e.g., *PR-8* and *PR-14*), cassava PR proteins were more closely related to poplar than rice, suggesting a divergence between monocots and eudicots. In contrast, some *PR* families, like *PR-6* and *PR-17*, showed no clear monocot/eudicot divergence. Finally, cassava-specific *PR* gene family expansions were found; this involved a total of 132 *PR* genes belonging to one of ten different *PR* gene families.

**Fig. 1:**
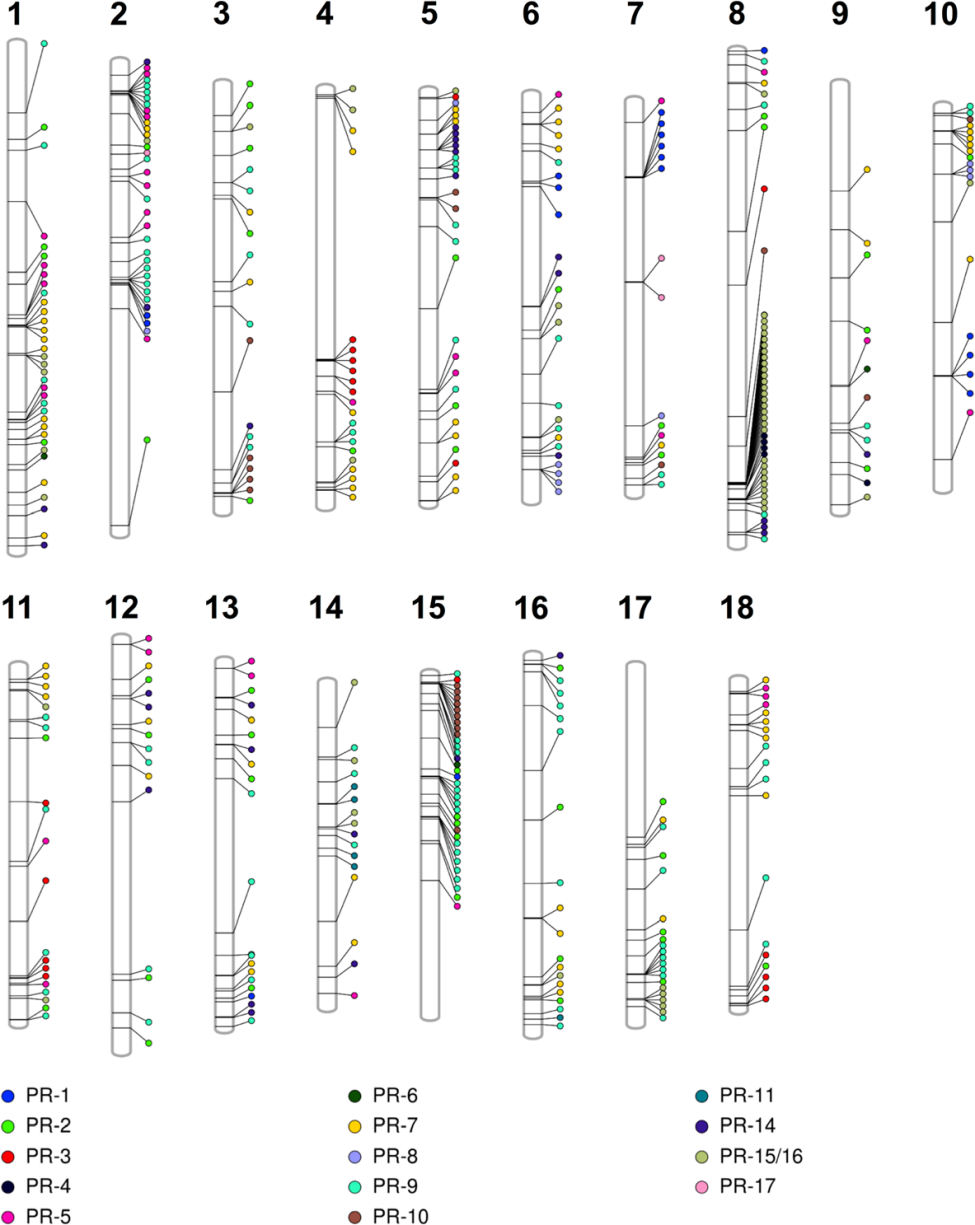
Physical locations of 435 *PR* genes along cassava chromosomes. *PR* families are color coded to reveal tandem arrays. Twelve *PR* genes have not been assigned to cassava chromosomes (Additional file 1: Table S1)

In addition, physical clustering of over 50% of the genes in *PR* families *1, 4, 5, 7, 8, 9, 10,* and *15/16* was observed (Fig. 1; Additional file 1: Table S2). Clustering was most prevalent in the *PR*-*15/16* family, where 29 of the 59 genes reside within three clusters on chromosome 8, with one cluster containing 20 genes. In contrast, all 50 *PR-2* family members were singletons, with no members belonging to a physical cluster (Fig. 1; Additional file 1: Table S2).

### Large *PR* families are downregulated after whitefly feeding

To characterize the response of *PR* genes to whitefly feeding, we analyzed the transcriptomes of four whitefly-susceptible cassava genotypes (COL2246, COL1468, 60444, and TME3) at 0, 1, 7, 14, and 22 days post-infestation (dpi) (Additional file 3: Tables S3-S6). We identified 167 *PR* genes that were differentially expressed (DEGs) during whitefly infestation in one or more genotypes at one or more time points (Table 2).

**Table 2:** Number of differentially regulated *PR* genes in whitefly-susceptible genotypes

*PR* gene family	COL2246		COL1468		60444		TME3		ALL		*PR* gene family size (# genes)
	Up	Down	Up	Down	Up	Down	Up	Down	Up	Down	
*1*	3	3	1	4	2	1	3	1	0	0	18
*2*	4	10	2	21	2	19	1	12	1	8	50
*3*	5	2	4	3	6	4	2	1	2	0	22
*4*	1	0	0	0	1	0	0	1	0	0	5
*5*	5	4	2	12	1	6	2	5	0	4	36
*6*	0	0	0	0	1	0	0	0	0	0	3
*7*	5	11	3	15	1	16	3	7	0	2	72
*8*	3	0	0	0	2	0	1	0	0	0	10
*9*	10	6	6	7	3	7	6	6	2	1	110
*10*	7	1	4	3	5	3	4	0	2	0	21
*11*	1	0	2	0	0	0	1	0	0	0	5
*14*	1	2	1	0	1	0	2	1	0	0	30
*15/16*	1	4	1	12	2	7	1	2	1	1	59
*17*	3	0	1	0	2	0	2	0	1	0	6
Total Number of DEGs	49	43	27	77	29	63	28	36	9	16	

In the large *PR* families *2, 7* and *15/16* with 50, 72 and 59 genes, respectively (Table 1), DEGs were mainly downregulated in the four cassava genotypes (Table 2). For example, the number of downregulated *PR-2* DEGs in the four genotypes was 2.5- to 12-fold higher than upregulated DEGs; a similar trend was observed in the *PR-7* family. In contrast, fewer *PR-15/16* genes were whitefly responsive, ranging from three DEGs in TME3 to 13 DEGs in COL1468. Notably, 12 of the 13 *PR-15/16* DEGs in COL1468 were downregulated. The largest *PR* family, *PR-9* with 110 genes (Table 1), had variable expression profiles. For example, there were 1.6-fold more up- than downregulated *PR-9* DEGs in COL2246. While at the other end of the spectrum, 60444 had 2.3-fold more down- than upregulated *PR-9* genes (Table 2). On the other hand, whitefly-upregulated DEGs were identified in most of the small *PR* families (*6, 8, 11*, and *17,* containing ten or fewer genes) but none were downregulated (Table 2).

### Timing of the response to whitefly varies among whitefly-susceptible genotypes

**Fig. 2:**
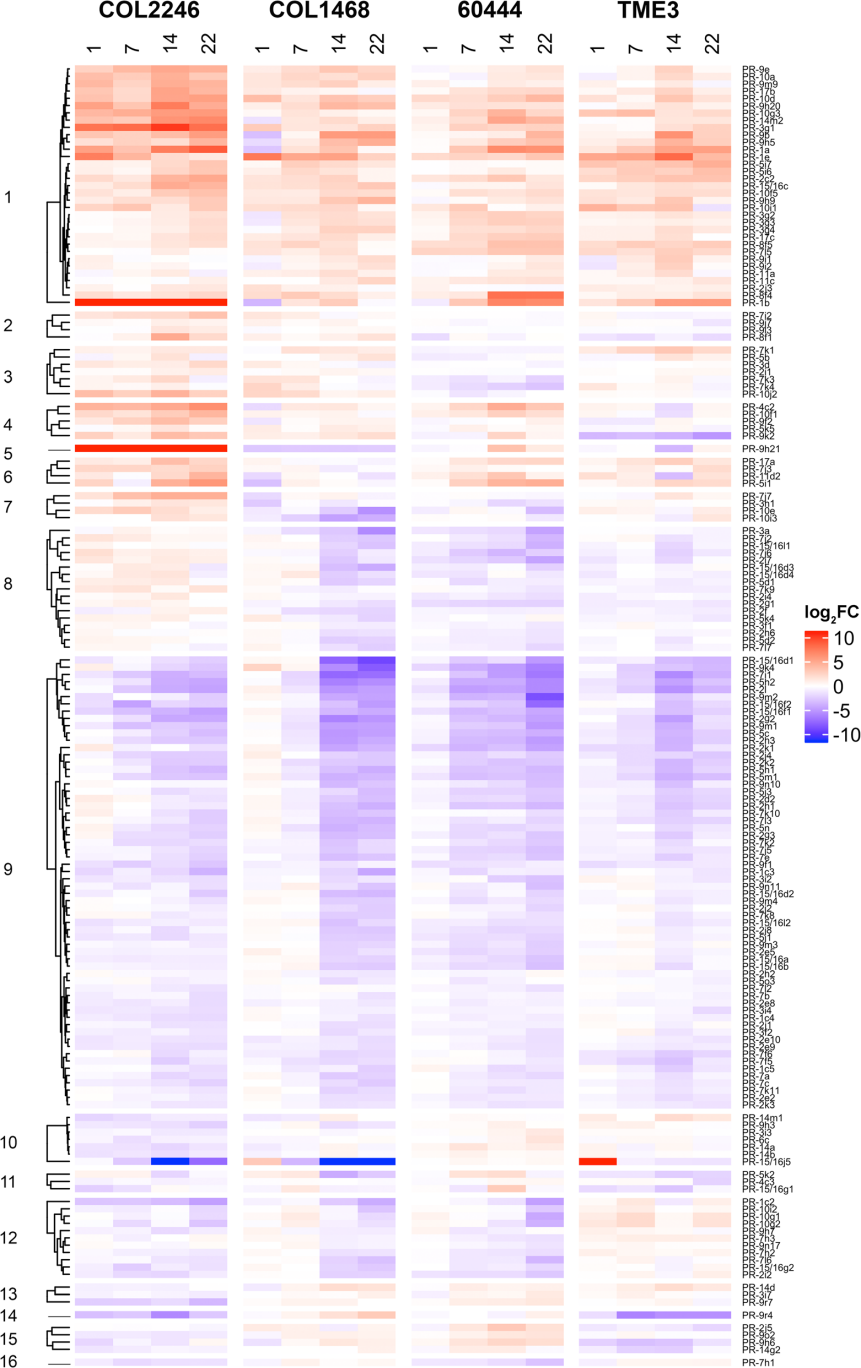
*PR* gene expression in whitefly-susceptible cassava genotypes during whitefly infestation. Heatmaps display DEGs in COL2246, COL1468, 60444, and/or TME3 during whitefly infestation. *PR* genes are grouped along the y-axis by expression patterns across genotypes as defined in Additional file 4: Table S15. Expression values are presented as log_2_FC values in comparison to 0 dpi

Heatmaps were used to define 16 temporal *PR* gene expression programs in response to whitefly feeding in the four genotypes (Fig. 2); for cluster definitions refer to Additional file 4: Table S15. Most striking, 57% of the 167 DEGs were similarly regulated among all genotypes, with 62 *PR* genes displaying negative trends (cluster 9) and 33 *PR* genes displaying positive trends (cluster 1) (Fig. 2; Additional file 4: Table S15). Cluster 9 was dominated by four *PR* families: *PR-2* (19 DEGs), *PR-7* (14 DEGs), *PR-5* (8 DEGs), and *PR-9* (8 DEGs). Of the 62 cluster 9 genes, 31, 55, 39, and 28 were downregulated at one or more time points in COL2246, COL1468, 60444, and TME3, respectively (Additional file 4: Table S15). A subset of these genes was downregulated in all four genotypes (16 DEGs) (Additional file 5); eight of which were *PR-2* genes (Table 2). Of the 33 *PR* genes in cluster 1, only nine were upregulated in all four genotypes (Additional file 6).

Cluster 1 and 9 genes displayed three temporal expression programs in response to whitefly infestation: early (1 and/or 7 dpi), late (14 and/or 22 dpi) and sustained (early and late). Few cluster 1 and 9 genes were differentially expressed at early time points. Only one early DEG in cluster 9 was identified (COL2246). For cluster 1, one early DEG was identified in COL2246 and 60444 and two early DEGs were found in COL1468. Finally, there are no early DEGs in either cluster 1 or 9 in TME3 (Additional files 6 and 7 b-e).

A prominent late phase of gene expression emerged in all genotypes, which engaged most of the cluster 1 and 9 *PR* genes and corresponded to the time of 2nd and 3rd instar feeding (Fig. 2). In all genotypes, most of the cluster 1 DEGs (39–78%) were upregulated by 14 dpi (Additional file 6). In contrast, the late phase of cluster 9 gene downregulation varied among the genotypes. For example, 42, 82 and 86% of the cluster 9 *PR* genes were downregulated by 14 dpi in COL2446, COL1468 and TME3, respectively. In 60444, this down-regulatory phase was further delayed, beginning at 22 dpi when 64% of cluster 9 *PR* genes were repressed (Additional file 5). The number of genes that displayed a sustained pattern of expression (DEGs at both early and late expression) varied among genotypes. While COL2246 and 60444 had 17 and 11 genes with sustained expression in cluster 1 or 9, respectively, (Fig. 2; Additional files 5 and 6), fewer genes in COL1468 and TME3 (4 and 3 genes, respectively) were regulated at both early and late time points.

The remaining 43% of whitefly-responsive *PR* genes (72 genes) exhibited divergent temporal responses among the genotypes (clusters 2–8 and 10–16). For example, 17 *PR* genes in cluster 8 were upregulated in COL2246 and downregulated in the other cassava genotypes. Additionally, cluster 12 genes were downregulated in COL2246, COL1468 and 60444. However, the timing of downregulation varied among genotypes, initiating later in COL1468 and 60444. Notably, ten of 60444’s 17 DEGs were in this cluster. In contrast, TME3’s cluster 12 genes had a slight positive trend (Fig. 2, Additional file 4: Table S15).

### Cassava *PR* genes are predominantly co-regulated by SA and JA

**Fig. 3:**
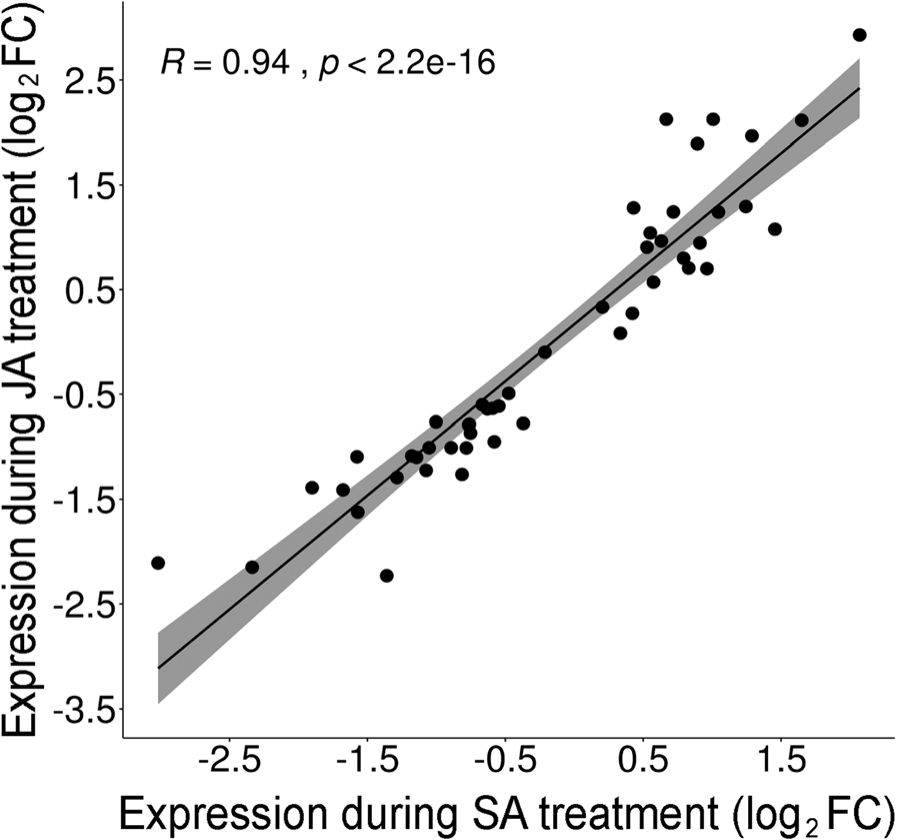
Correlation of SA/JA co-regulated *PR* genes. Average log_2_FC of DEGs in SA versus JA treatments for *PR* genes designated as SA/JA co-regulated (defined in Additional file 7). Pearson correlation value, *p*-value and a 95% confidence interval (grey) are provided

To understand the roles of the two major plant-defense hormones (SA and JA) in regulating *PR* genes, we determined the transcriptomes of COL2246 at eight time points (0, 0.5, 1, 2, 4, 8, 12, and 24 h) after SA and JA treatments (Additional file 3: Tables S7-S14). Hormone-responsive *PR* genes (103 DEGs out of the 447 *PR* genes) were organized into one of four hormone-expression programs: 1) SA-regulated (10 DEGs), 2) JA-regulated (42 DEGs), 3) co-regulated by SA and JA (49 DEGs), or 4) reciprocally regulated by SA and JA (2 DEGs) (Table 3; Additional file 7). *PR* families *2*, *5*, *7,* and *9* made up 65% of hormone-responsive DEGs and were mainly SA/JA co-regulated or JA-regulated. There was a very strong positive correlation (R = 0.94, *p* = 2.2e^− 16^) between SA and JA expression levels for SA/JA co-regulated DEGs (Fig. 3; Table 3; Additional file 7). Of the genes defined as solely SA- or JA-regulated, 81% exhibited similar expression levels in response to both treatments, but only met the statistical criteria to be designated as DEGs in one treatment (Additional file 7). Furthermore, while *PR* genes are useful markers to follow the activation of the SA (*PR-1*, *− 2* and *− 5*) and JA (*PR-3* and *-4*) pathways in Arabidopsis-pest/pathogen interactions [21], we were unable to identify any *PR* gene that could distinguish activation of only the SA or JA pathway.

**Table 3:** Hormone-regulated *PR* genes^a^

	Number of hormone-regulated DEGs				
*PR* Gene Family	SA	JA	SA/JA co-regulated^b^	SA/JA reciprocally regulated	Number of hormone-responsive DEGs per *PR* family
1	0	1	2	0	3
2	1	4	10	1	16
3	2	3	0	0	5
4	0	2	0	0	2
5	1	4	5	0	10
6	1	0	1	0	2
7	2	8	14	0	24
8	0	0	1	0	1
9	1	7	8	1	17
10	1	4	2	0	7
11	0	2	1	0	3
14	0	0	2	0	2
15/16	1	6	1	0	8
17	0	1	2	0	3
Number of hormone-responsive DEGs across *PR* families	10	42	49	2	103

^a^For identities of hormone-regulated *PR* genes, see Additional file 8

^b^SA and JA co-regulated genes are defined as genes whose RNAs are either up- or down-regulated by both hormones

**Fig. 4:**
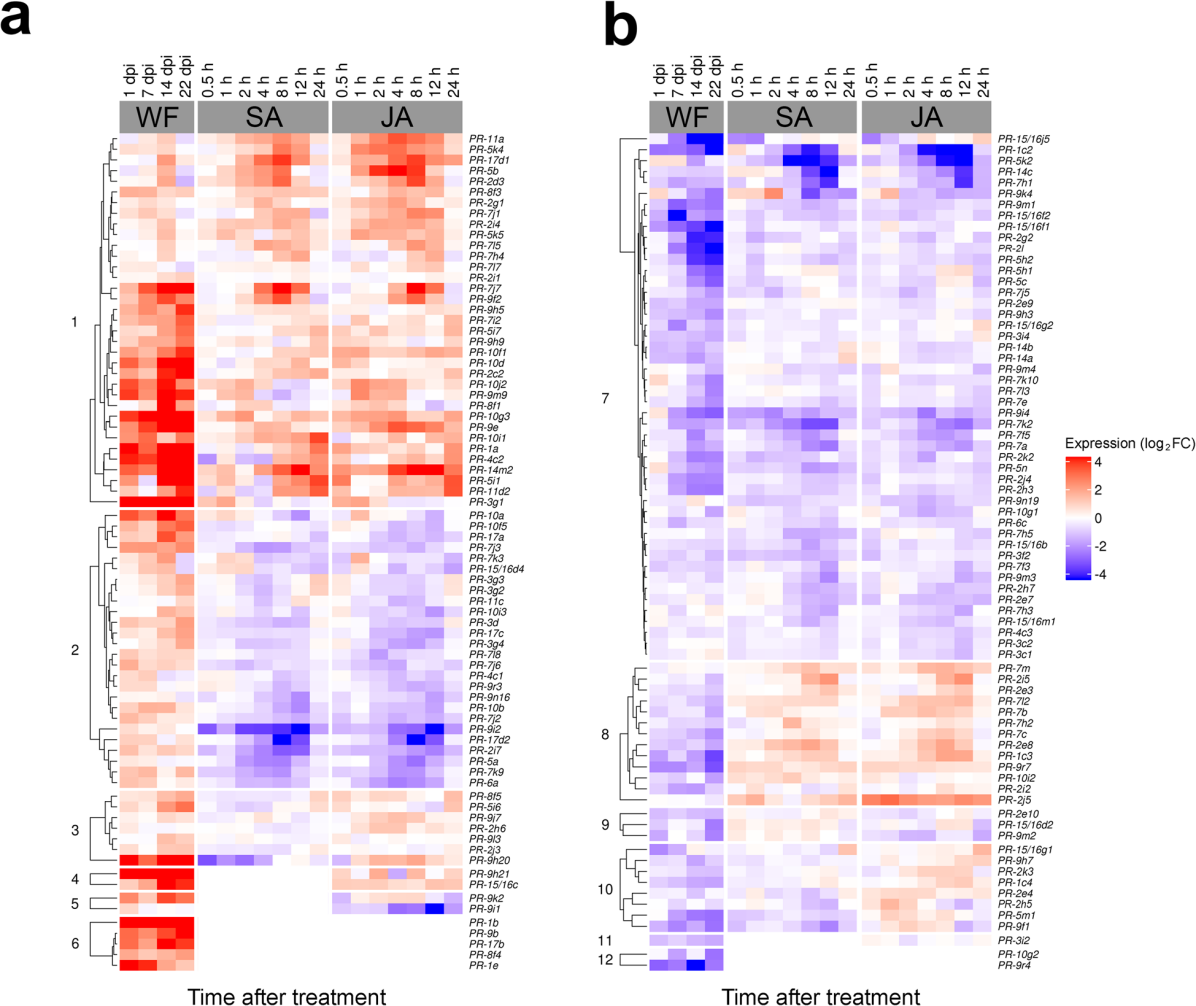
*PR* gene expression in COL2446 in response to whitefly infestation, SA and JA. Heatmaps were organized along the y-axis to group *PR* genes with positive **a** or negative **b** expression values in whitefly (WF)-infested COL2246. Expression after SA and JA treatments are shown. log_2_FC values relative to 0 dpi for whitefly-infested samples and 0 h for hormone treatments. Heatmap groups 1–12 are defined in Additional file 4: Table S16

To characterize the hormone regulation of whitefly-responsive *PR* genes in COL2246, we integrated whitefly, SA and JA transcriptome data (Fig. 4). Among the 208 *PR* genes detected during whitefly infestation of COL2246, 152 genes were DEGs in whitefly, SA and/or JA treatments of COL2246 (Fig. 4; Additional file 3: Table S3, S7, and S11). While plant defenses typically enact a predominant SA or JA response in Arabidopsis [34, 35], 122 (80%) of the 152 genes were co-expressed during SA and JA treatments (clusters 1, 2, 7, and 8). Notably, there were no whitefly-responsive *PR* genes that were solely detected after SA treatment (Fig. 4).

In COL2246, these hormone-responsive *PR* genes displayed three temporal expression programs (early, late and sustained) after whitefly infestation (Fig. 4). While only four whitefly-regulated DEGs followed an early expression program, 24 exhibited sustained regulation and 64 were late-regulated (Additional file 8). Genes with sustained regulation displayed more positive (40%) than negative (16%) expression trends in response to whiteflies, SA and JA (clusters 1 and 7, respectively) (Fig. 4; Additional file 8). In contrast, for the late-regulated genes, negative expression trends were more frequent (31%) than positive (23%) trends in all three treatments (clusters 7 and 1, respectively) (Fig. 4; Additional file 8).

### qRT-PCR validation of RNA-sequencing data

**Fig. 5:**
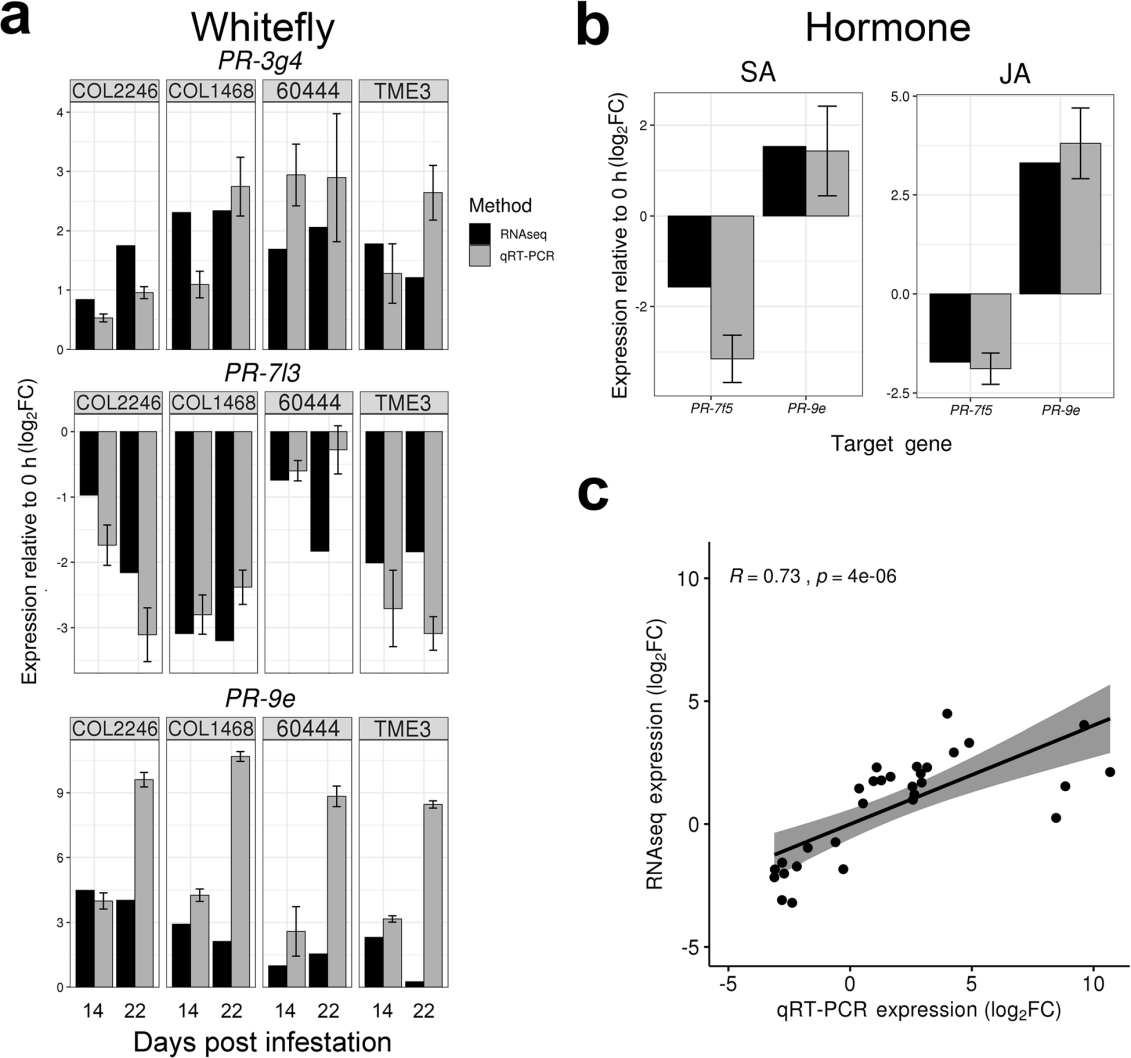
qRT-PCR validation of *PR* transcript levels. **a-b** Relative transcript levels (log_2_FC) of up- and down-regulated *PR* genes during whitefly infestation of COL2246, COL1468, 60444, and TME3 **a** and after SA and JA treatments of COL2246 **b** as determined by qRT-PCR and RNAseq. **c** Pearson correlation of transcript levels in **a** and **b** by qRT-PCR versus RNAseq

To confirm expression values obtained in silico, transcript levels of selected whitefly- or hormone-responsive DEGs were assessed by qRT-PCR (Fig. 5). Upregulation of *PR-3g4* and *PR-9e* and downregulation of *PR-7l3* at both 14- and 22-dpi after whitefly infestation was confirmed (Fig. 5a). Similarly, *PR-9e* upregulation and *PR-7f5* downregulation after 4-h SA and JA treatments was confirmed (Fig. 5b). In many cases, transcript fold-changes determined by qRT-PCR exceeded those measured by RNA-seq (Fig. 5a and b). Nevertheless, expression values for *PR* genes obtained by qRT-PCR versus RNA-seq exhibited a strong positive correlation (R = 0.73; *p* = 4.0E^− 06^), validating our in silico expression values in vivo (Fig. 5c).

### Comparison of *PR* family responses to a spectrum of biotic stressors

**Fig. 6:**
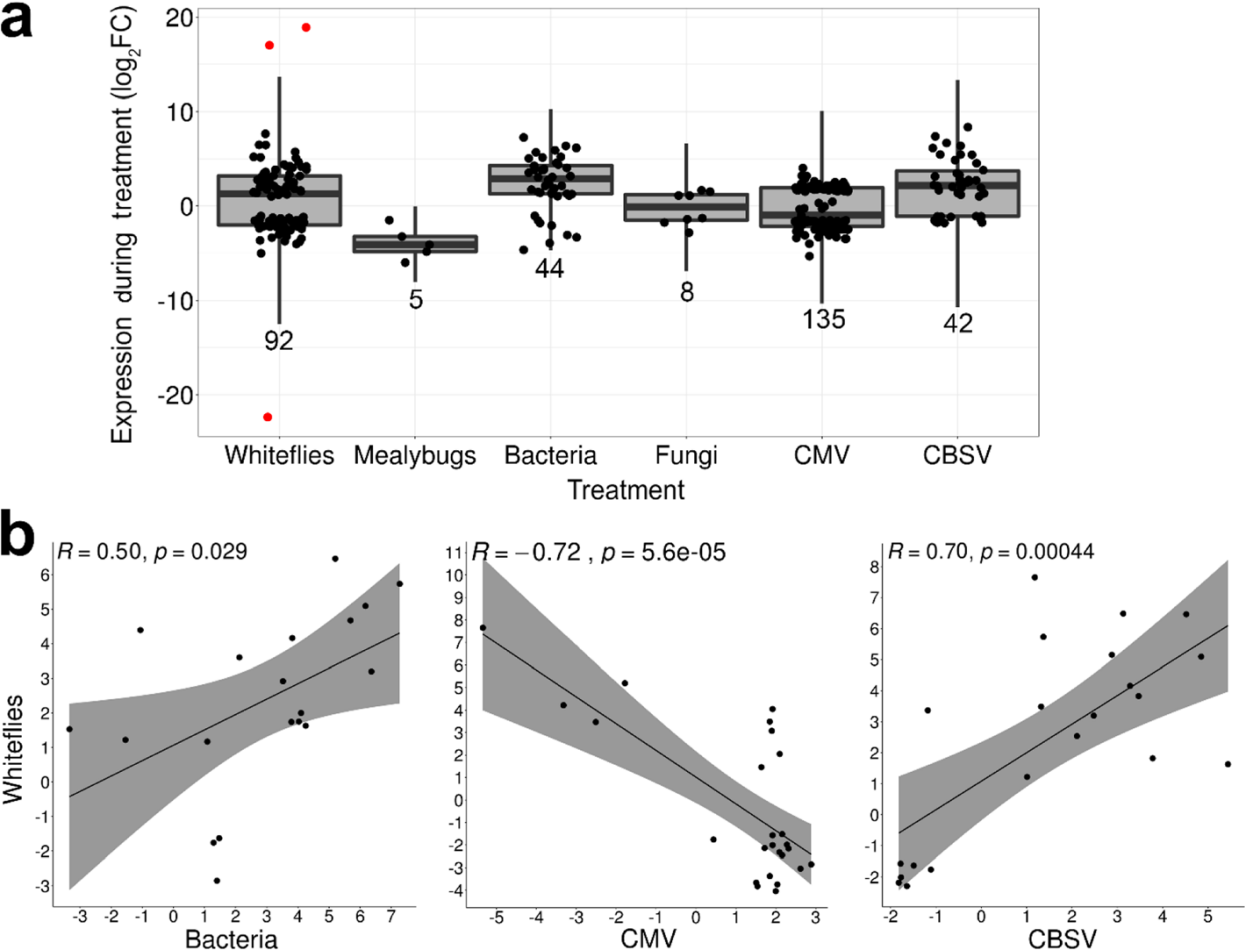
Correlation of cassava responses to whiteflies with other biotic stresses. **a** Box-and-whisker plots overlaid with all data points displaying mean *PR* gene responses to treatments. Whiskers indicate the interquartile range multiplied by two. Total number of genes for each treatment is below each plot. Outliers (red) were identified and removed prior to correlation analyses in panel **b**. **b** Pearson correlation analyses of DEGs responding to whiteflies vs. bacteria (19 genes), CMV (25 genes) or CBSV (21 genes) were performed. Only four genes were identified in the whitefly-mealybug and whitefly-fungus interactions; therefore, these interactions were not included in these analyses

To more broadly define the responses of cassava’s *PR* genes in pathogen and pest interactions, we compared *PR* gene expression programs to whiteflies (*A. socialis*) with five other pathogens/pests: cassava mealybugs (*Phenacoccus manihoti*), bacteria (*X. axonopodis*), fungi (*C. gloeosporioides*), and viruses (South African CMV and CBSV) [26, 28–30, 33] (Additional file 9; Additional file 1: Table S1; Additional file 3: Tables S3, S7 and S11). Each interaction elicited a different number of DEGs; therefore, to facilitate comparisons, the percent of DEGs from each *PR* family that responded to each biotic stress was determined (Table 4; Fig. 6a). We found that *PR* families with roles in pathogen cell wall degradation (*PR-2*, *PR-5* and *PR-7*), as well as host cell wall fortification (*PR-9*) were most responsive to biotic stress, representing 10–26% of the *PR* genes responding to any of the examined stresses.

**Table 4:** *PR* gene family response (DEGs) to six biotic stresses^a^

	Percent of response attributed to a *PR* gene family^b^						
*PR* Gene Family	whiteflies	mealybugs	bacteria	fungi	virus (CMV)	virus (CBSV)	Percent of *PR* gene family responsive to one or more stresses
1	7	20	7	25	1	10	4
2	15	0	14	0	11	10	13
3	8	0	7	13	2	10	6
4	1	0	2	0	1	2	1
5	10	60	11	13	9	5	10
6	0	0	0	0	1	0	0
7	17	0	9	13	17	7	16
8	3	0	7	0	2	0	3
9	17	0	23	38	35	33	26
10	9	0	7	0	4	5	6
11	1	0	0	0	0	2	1
14	3	20	7	0	7	10	5
15/16	5	0	5	0	10	5	8
17	3	0	2	0	0	2	1
# of *PR* DEGs	92	5	44	8	135	42	326

^a^For identities *PR* genes differentially expressed during the biotic stresses, see Additional file 11. For study information, see Methods

^b^Percents are rounded to the nearest integer

In these interactions, 38–75% of differentially expressed *PR* genes responsive to whiteflies, bacteria or fungi were regulated by SA and/or JA (Additional files 10, 11, 12, 13 and 14; Additional file 15: Tables S17-S22) and a majority (57–67%) of these genes were in the *PR*-*5*, *PR-7* or *PR-9* families (Additional file 11: Figure S19; Additional file 12: Figure S21; Additional file 13: Figure S23; Additional file 15: Tables S17, S19 and S20). In contrast, most genes (79–80%) regulated by mealybugs, CMV or CBSV were not responsive to either hormone (Additional files 12, 13, 14, 15 and 16, Additional file 15: Tables S18, S21 and S22).

Pearson correlation analyses were used to compare cassava’s *PR* gene response to whiteflies, bacteria, CMV, and CBSV (see Methods) (Fig. 6b). We identified a moderate positive correlation between whiteflies and bacteria (R = 0.50; *p* = 2.9E^− 02^) and a strong positive correlation between whiteflies and CBSV (R = 0.70; *p* = 4.4E^− 04^). In contrast, responses to whiteflies and CMV were dissimilar, showing a strong negative correlation (R = − 0.72; *p* = 5.6E^− 05^) (Fig. 6b). Correlations between whitefly and bacteria/CBSV and between whitefly and CMV were associated with 30 and 19 DEGs, respectively (Additional file 16: Tables S23 and S24).

### Integration of defense transcriptomes and cassava *PR* gene phylogenies

To visualize cassava *PR* family responses to biotic stress, SA and JA, *PR* clades identified in cassava *PR* phylogenetic trees were used to order heatmaps (Additional files 12, 13, 14, 15 and 16). We integrated these data with the *PR* family expansions identified in the cassava, poplar and rice PR phylogenetic trees (Additional file 2). Of the 132 *PR* genes in cassava expansions, 43 were DEGs in response to at least one biotic interaction and/or defense hormone (Additional files 12, 13, 14, 15 and 16).

Several expansions in *PR* families *2, 3, 9, 10, 14,* and *15/16* were associated with responsiveness to whiteflies. For example, in the *PR-2* gene family, all three genes (*PR-2e8, PR-2e9* and *PR-2e10*) in the *PR-2e* expansion were whitefly-downregulated (Additional file 10: Figure S16). *PR-2e8* was also upregulated in response to SA and JA. The *PR-3g* expansion (*PR-3g2, PR-3g3* and *PR-3g4*) (Additional file 10: Figure S17) was whitefly-upregulated. Although *PR-3g3* and *PR-3g4* were located in tandem on chromosome 11, they had distinct responses to other pathogens. *PR-3g4* was upregulated by whiteflies, fungi and bacteria, while *PR-3g3* and *PR-3g2* were strongly upregulated by whiteflies and CBSV. (Additional file 1: Table S1; Additional file 2).

While all genes in the *PR-2e* and *PR-3g* expansions were whitefly-responsive, within other cassava-specific expansions in *PR* families *9*, *10*, *14*, and *15/16* whitefly responsiveness was detected in a subset of the genes in the expansion. For example, in the 20 gene *PR-15/16j* clade on chromosome 8, *PR-15/16j5* was the only gene regulated by any biotic stress and it was strongly downregulated by whiteflies (Additional file 14: Figure S27).

Genes within 13 cassava expansions from *PR* families *1, 5, 7, 8,* and *9* did not contain whitefly-responsive DEGs (Additional file 10: Figure S15; Additional file 11: Figure S19; Additional file 12: Figures S21 and S22; Additional file 13: Figure S23). Strikingly, when cassava expansions were examined collectively numerous genes (34 genes) that responded to CMV and/or CBSV were identified and 16 of these genes belonged to the *PR-9* family.

These integrative analyses also highlighted that the *PR* families *1*, *14* and *15/16* had a high proportion (39–41%) of *PR*-*Like* genes, which were not differentially expressed in response to any stress or hormone (Additional file 1: Table S1). In particular, the expansions of the *PR-1d*, *PR-14e*, *PR-15/16i*, and *PR-15/16j* clades were rich in *PR-Like* genes (Additional file 10: Figure S15; Additional file 14: Figure S26 and S27), suggesting their recent evolution is not associated with the biotic stresses presented here. However, many of these genes are expressed in embryonic structures, fibrous roots, or the root apical meristem (Additional file 17), suggesting they may function in different organs, during growth and development, or during biotic or abiotic interactions that were not included in our study.

## Discussion

### *PR* family composition and organization in cassava

In cassava and three other plant species, we showed that *PR* genes exist as multigene families, as previously described for cacao (*Theobroma cacao*), pine (*Pinus spp.*) and barrel medic (*Medicago truncatula*) [22–24]. While *PR* family numbers differed from other published *PR* gene numbers due to our methods for gene identification [22], overall *PR* family sizes were similar in the four species analyzed, with a few exceptions. Similar to cacao [22], the *PR-10* family was expanded in both cassava and poplar, relative to rice and Arabidopsis. In addition, PR-12 and PR-13 proteins were not detected in the cassava or poplar genomes. PR-12 was also absent in two pine species [23] and PR-13 has been previously described only in monocots and in the Brassicaceae [22, 36].

As described by Fister et al. [22] for cacao, Arabidopsis*, Brachypodium distachyon,* rice, poplar, and *Vitis vinifera*, many genes (51%) within each cassava *PR* family were clustered. As in cacao, *PR-10* and *PR-15/16* families had the largest gene clusters. In contrast, while *PR-2* and *PR-6* genes were clustered in the cacao genome [22], cassava’s *PR-2* and *PR-6* genes occurred as singletons. Tandem organization of *PR* genes has also been described for *PR-12* in Arabidopsis [37], *PR-7* in tomato [38] and the *PR-10* family in grape [39].

As plants and their attackers are continually coevolving, such evolutionary pressures commonly lead to the expansion and diversification of host-plant defense gene families [15]. This phenomenon is well-documented in resistance gene families, such as the NLRs, and can also be found in various defense gene families [40, 41]. These principles also appear to apply to cassava’s *PR* gene families. By integrating our transcriptomic and phylogenetic data sets, we observed multiple instances of cassava *PR* family expansions associated with responses to whiteflies and other biotic stresses that may indicate selection for new functions for these paralogs.

For example, expansions within the *PR-2e* and *PR-3g* clades were associated with whitefly downregulation and whitefly/microbe upregulation, respectively. As PR-2 (β-1,3-endoglucanases) and PR-3 (chitinases) proteins are commonly involved in responses to bacteria/fungi and have antimicrobial activities [21, 42–44], it is possible that whiteflies and microbes produce similar pressures for the evolution of paralogs in *PR* gene family expansions. Whitefly feeding produces little cellular damage similar to biotrophic bacteria/fungi, and whitefly stylet movement may be perceived as similar to bacterial spread or fungal hyphae movement through the apoplast [45]. Alternatively, endosymbionts and/or their gene products present in whitefly saliva [46] or chitin derived from whitefly stylets or exoskeletons during molting may be perceived as bacteria/fungi-like triggers for regulation of *PR* and other defense genes [47].

### Cassava’s *PR* gene responses to whitefly and other pathogens

To date, there is limited information about cassava’s *PR* gene regulation and function. In 2006, Antony and Palaniswami [48] showed that PR activities (β-1,3-glucanase, peroxidase and chitinase) increased after *B. tabaci* infestation. In addition, using yeast two-hybrid assays, Román et al. [49] constructed a PR protein-interaction network that is deployed during *Xanthomonas* infection. We discovered that 37% of the differentially expressed *PRs* were downregulated (62 genes in cluster 9, Fig. 3). This large number of downregulated genes is a surprising result as it contrasts with the definition of *PR* genes as being upregulated after pathogen or pest attack [21]. Also, this large-scale downregulation of cassava *PR* genes after whitefly infestation does not align with previous studies in cacao in response to pathogens *Phytophthora palmivora* and *Colletotrichum theobromicola* [22].

Cassava’s unique *PR* gene regulatory programs may be due to one or more factors. First, some of the cassava *PR* homologs identified in this study, while sharing sequence identity with other land plant PR proteins, may have been recruited for new functions to survive stressful environments or to play a role in growth and development. Second, as the size of the *PR* families increases, the resulting paralogs are functionally redundant and some can be recruited to new roles without compromising cassava’s defense. Such gene family functional evolution may have occurred in cassava. In fact, the PR network established by Román et al. [49] showed that many of the cassava proteins interacting with PRs were associated with abiotic stress or metabolic responses and were not implicated in defense. Additionally, PR proteins regulate cell proliferation/differentiation in tobacco [50] or can rescue of somatic embryos in carrot [51]. These new functional roles for PRs are also consistent with the expression of many cassava *PR* genes during somatic embryogenesis, as well as in shoots and roots.

Finally, the downregulation of *PR* genes could be due to whitefly effectors that actively suppress plant immunity [45, 46]. Notably, in Arabidopsis, whiteflies promote SA-regulated *PR* gene expression, rather than suppress it [52, 53]. Alternatively, cassava’s regulatory circuitry of *PR* genes may be significantly different than reported in other species to date. Indeed, our analysis of published data for five other cassava-pathogen interactions indicated that *PR* gene downregulation was common in these other cassava-pathosystems (Fig. 6) [26–33].

### Hormone regulation of whitefly-responsive *PR* genes

Plant defenses are commonly associated with hormone programs specific to the attacker and often controlled by SA or JA in response to biotrophs and necrotrophs/wounding, respectively [54]. In our analysis of cassava *PR* genes, coordinate SA and JA responses characterize over 90% of the interaction between whitefly-susceptible cassava and whiteflies. In the context of plant-hemipteran interactions, there are a few instances where SA and JA responses are activated concurrently, including the pepper-whitefly interaction [55]. However more generally, synergistic SA and JA responses have been found to be associated with resistance to certain biotic stressors [56].

While studies defining the hormone response of the full complement of *PR* families in other species have not been reported, transcriptome studies in Arabidopsis, rice and sorghum assessing response to SA and JA reported that 20–50% of all genes responsive to SA and/or JA exhibited coordinate responses [57–59]. This variation in global SA and JA expression programs among plant species points to the need for species-specific definitions of *PR* genes used as defense markers.

### Cassava *PR* families *2*, *5*, *7*, and *9* are most responsive to biotic stress and hormones

Across the many cassava-pest/pathogen/hormone interactions examined in this study, *PR* families with roles in pathogen cell wall degradation (*PR-2*, *PR-5* and *PR-7*), as well as host cell wall fortification (*PR-9*) were the predominant families responding to whiteflies (*A. socialis*), SA, JA, and most other biotic stresses (mealybugs (*P. manihoti*), fungi (*C. gloeosporioides*), bacteria (*X. axonopodis*), CMV, and CBSV) [26, 28–30, 33]. PR-2 and PR-5 proteins have been reported as β-1,3-endoglucanases and β-glucan-binding proteins, respectively, with roles in pathogen membrane degradation/permeabilization [60, 61]. PR-7 proteins are endoproteinases and have proposed roles in aiding fungal cell wall degradation [62]. PR-9s are lignin-forming peroxidases that may reinforce the cell wall by catalyzing lignification and preventing pathogen penetration [21, 63].

In other global *PR* family analyses, *PR* families *2*, *5* and *9* were also well-represented in the response of poplar to the fungus *Melampsora larici-populina* [25] and in cacao interactions with the oomycete *Phytophthora palmivora* and the fungus *Colletotrichum theobromicola* [22]. Previous studies of plant responses to hemipterans have followed the regulation of only well-documented *PR* sentinel genes, or have not adequately analyzed *PR* gene expression profiles within transcriptome studies [64]. Among the most responsive *PR* families identified in our study (*PR-2*, *PR-5*, *PR-7*, and *PR-9*), Arabidopsis *PR-2* and *PR-5* [52, 53] and tomato *PR-2* [65–67] have been shown to be induced in response to whitefly infestation. Similarly, *PR-2* is induced after aphid feeding on sorghum and Arabidopsis [59, 68].

Among the biotic stresses examined, a large portion (38–75%) of *PR* genes responsive to whiteflies (*A. socialis*), bacteria (*X. axonopodis*) or fungi (*C. gloeosporioides*) were regulated by SA and JA, while these hormones were unlikely to play a major role in regulating cassava’s response to viruses. Other signals may be responsible for their regulation.

### *PR* gene responses to whiteflies are more similar to CBSV than to CMV

Cassava’s *PR* gene responses to whiteflies were positively correlated with its response to bacteria (*X. axonopodis*) and CBSV but negatively correlated with its responses to CMV [28, 30, 33]. The distinct *PR* gene responses to CMV and CBSV could be due to the different mechanisms of inoculation used in each study [30, 33] or the different viral replication strategies employed by these two viruses [69]. Alternatively, *PR* gene responses to the two viruses could be reflective of the different modes of CMV and CBSV acquisition and transmission by their whitefly vector [69–71]. The biological significance and molecular mechanisms that underlie the distinct *PR* gene signatures to CBSV and CMV, and their correlations with whitefly *PR* gene expression programs remain to be discovered.

## Conclusions

In this study, we provided the first genome-wide identification of *PR* gene families in cassava and characterized cassava’s transcriptome response to whiteflies, SA, JA, and other biotic stressors. Utilizing four susceptible cassava genotypes with diverse genetic backgrounds, we identified core transcriptome responses to whitefly infestation. Surprisingly, many *PR* genes, which are canonically “inducible”, were downregulated in response to whitefly and other biotic stressors, suggesting novel functions of such *PR* genes, or, unusual *PR* gene regulation specific to cassava. Nevertheless, the gene expression programs identified for *PR* families *2*, *5*, *7*, and *9* as predominating the response to whiteflies, SA, JA, and most other biotic stresses, suggests their functionalities (pathogen cell wall degradation and host cell wall reinforcement) play an important role in cassava defense responses.

Definition of the SA- and JA-dependent transcriptomes of cassava revealed that whitefly, bacteria and fungi *PR* gene responses are largely coordinately regulated by SA and JA. Correlation and phylogenetic analyses uncovered additional similarities in whitefly/microbe responses with positive correlations in *PR* DEGs when whitefly responses were compared to bacteria and CBSV responses. Comparison of PR family composition among plant species revealed cassava-specific expansions within PR clades. Notably, clades *PR-2e* and *PR-3g* contain genes within cassava-specific expansions associated with whitefly/microbe responses. Together, we suggest that *PR* gene responses may be comparable among whiteflies and certain microbes due to similar perception of the whitefly stylet (chitin, apoplast movement) or of whitefly saliva components (elicitors, endosymbionts).

Collectively, our study contributes to our understanding of the genetic basis of cassava’s response to various yield-threatening pests/pathogens, which is essential for implementing a multitude of different control strategies. We additionally define the SA- and JA-dependent transcriptomes of cassava, which will facilitate characterization of cassava-pest/pathogen interactions. Our integrated data set of *PR* gene stress/hormone expression programs, physical distributions and phylogenetic relationships will also serve as a useful tool for the cassava defense community. Finally, it is hoped that the genome-wide analysis of cassava’s *PR* gene families emphasizes the need for evaluating the *PR* gene regulatory programs in other crops to develop an understanding of the utility of *PR* genes as defense sentinels.

## Methods

### Plant growth

Shoot tips from in vitro grown *Manihot esculenta* genotypes (COL2246, COL1468 (CMC40), 60444 (TMS60444/NGA11), and TME3) in the CIAT culture collection were excised and grown in 17 N rooting medium for 30 days. Plants were then sown in 2-L pots with sterile soil with a ratio of 1:3 sand to black soil (no clay topsoil). Plants were grown in a glasshouse with temperatures ranging from 24 to 28 °C under a long-day light cycle (16-h light/ 8-h dark). Sixty days after sowing, plants were used for hormone treatments or whitefly infestation experiments.

### Mass rearing of *Aleurotrachelus socialis* and whitefly bioassays

The *Aleurotrachelus socialis* colony was raised on *Manihot esculenta* var. COL1468 as previously described by Bellotti and Arias [5]. For the whitefly-infestation experiments, four whitefly-susceptible cassava genotypes (COL2246, COL1468, 60444, and TME3) were used [72, 73]. Each three-month-old plant was put into an individual mesh cage (1-m height × 30-cm diameter) in a glasshouse. Infestations were initiated by the release of 100 male and 100 female adults of *A. socialis* into each cage. When the adult whiteflies were removed at 3 dpi, the two youngest infested leaves, which are preferred by whiteflies for feeding and egg deposition, were tagged for future collection. Three biological replicates were used for each genotype. Infested plants were placed in a random design instructed by a factorial arrangement. In order to capture the effect of each life stage of the whitefly on the cassava plants, the sample collection time points were chosen to represent landmarks during the *A. socialis* life cycle [5]. Samples were harvested at: 0 h post-infestation (hpi), 1 dpi (adult feeding and egg deposition), 7 dpi (1st instar feeding), 14 dpi (2nd and 3rd instar feeding), and 22 dpi (4th instar feeding and emergence). After collection, leaves were frozen in liquid nitrogen and stored at − 80 °C until use.

### Hormone treatments

One day prior to hormone treatments, three-month-old COL2246 plants were moved from the glasshouse into growth chambers with a 16-h light/ 8-h dark cycle and a 24–28 °C temperature range. Cassava leaves were sprayed to saturation with salicylic acid (200 μM SA, 0.1% EtOH, 0.01% Tween 20) and methyl jasmonate (7.5 mM MeJA, 0.1% EtOH, 0.01% Tween 20) [74, 75]. Treatments were performed in growth chambers in different rooms. The 0-h sample was collected at 9 AM. All leaves (4–6 per plant) were sprayed with SA or JA until saturation and were harvested at 0.5, 1, 2, 4, 8, 12, and 24 h post treatment by excising the leaf blade. Tissue was frozen in liquid nitrogen and stored at − 80 °C until use. This experiment was conducted three times (three biological replicates).

### PR protein phylogenetic trees, gene nomenclature and genome location

For the 17 PR protein families defined by van Loon et al. [21], founder PR protein sequences were used as queries to identify cassava PR proteins. Cassava, poplar, rice, and Arabidopsis PR proteins were obtained from Phytozome (JGI) and Ensembl Plants using BLASTP and Hidden Markov Model searches [76], respectively. Percent identity and E-values of the cassava PR proteins are provided (Additional file 1: Table S1). The Pfam database [77] was used to identify conserved protein domains that distinguish each PR family. The PR proteins that lacked canonical PR domains (20 of 447 proteins) were removed prior to alignment with ClustalW. The resulting alignments were manually curated and neighbor-joining phylogenetic trees were constructed using Geneious version 11.1.2 [78]. Bootstrapping was performed with 1000 replications and bootstrap values are shown only for branches with 50% or higher bootstrap support. Cassava-specific *PR* gene family expansions were defined as cassava clades that contained three or more cassava paralogs.

Proteins within each cassava *PR* family were used to construct family phylogenetic trees. The *PR* genes were named according to their phylogenetic relationships in *Manihot esculenta* (Additional files 12, 13, 14, 15 and 16). Within a family, genes were assigned a letter indicating their clade (i.e., *PR-1d*). *PR* genes that were not differentially expressed during pest/pathogen treatment were designated as *PR-Like* genes (i.e., *PR-1dL*) (Additional file 9). *PR* and *PR-L* genes within a clade were numbered sequentially (Additional file 1: Table S1). *PR* genes assigned to cassava chromosomes 1 to 18 were visualized using the program PhenoGram [79].

### RNA extraction and quality assessment

Total RNA was extracted using the methodology described by Behnam et al. [80]. RNA was quantified using a Nanodrop® ND-1000 spectrophotometer (Thermo Scientific, Wilmington, Delaware, USA). RNA quality was assessed by absorbance ratios, denaturing agarose gels and an Agilent 2100 Bioanalyzer (Agilent Technologies, Palo Alto, CA). RIN values were above 6.00. RNAs were treated with 20 μL of RNAstable® (Biomatrica, San Diego, CA) and then dried using the Speedvac Concentrator™ (Eppendorf™) for 1 h at room temperature. RNA quantity and integrity was confirmed prior to RNA-seq library construction using the Nanodrop® ND-1000 spectrophotometer and 1% denaturing agarose gels, respectively.

### cDNA library preparation, sequencing and bioinformatics analyses

Strand-specific cDNA libraries were prepared following the protocol of Wang et al. [81] with two changes. The reverse transcriptase used was Superscript III (Invitrogen, Carlsbad, CA) and the high-fidelity DNA Polymerase was KAPA HiFi Hot start (KAPA Biosystems, Wilmington, MA). Universal and barcoded primers were purchased from Integrated DNA Technologies (Coralville, IA).

cDNA libraries generated from whitefly-infestation experiments were sequenced on the Illumina HiSeq2500 platform (single-end 50-bp reads) or on the Illumina NextSeq500 platform (single-end 75-bp reads) at the UCR Institute for Integrative Genome Biology Genomics Core. Seventy-five-bp reads were trimmed to 50 bp to allow valid comparisons of all libraries. Libraries from SA and MeJA experiments were sequenced on the NextSeq500 platform (single-end 75-bp reads). For each infestation or treatment time point, the three biological replicates were used to construct libraries. Libraries were multiplexed (12–13 libraries/lane) and sequenced resulting in ~ 25–45 million reads per library. Using total read counts, Pearson correlation values ranging from 0.70–1.00 and from 0.89–0.99 were obtained among biological replicates for whitefly and hormone treatments, respectively, confirming their reproducibility (Additional file 18). After trimming and filtering the fastq files, reads were aligned against the *Manihot esculenta* genome version 6.1 at Phytozome [82], using Bowtie2/2.2.5 and Tophat 2.0.14. The subsequent analyses of the sequences were made following the systemPipeR pipeline [83]. Genes with an average of 20 reads or less across a treatment time course were not included in the DEG analysis. DESeq2 was used to determine DEGs, defined as having |log_2_FC| > 1 and FDR ≤ 5%.

Heatmaps for whitefly infestation studies and hormone treatment studies were organized by defined expression programs (Additional file 4: Tables S15-S16) and hierarchical clustering along the y-axis and were constructed using the R package ComplexHeatmap [84]. Venn diagrams used to visualize DEGs were generated using the online program Venny [85]. Raw data are provided in Additional file 3.

Data from published transcriptome studies investigating five additional cassava-pathogen/pest interactions were used for comparison to our transcriptome data sets for COL2246. It should be noted that these studies were performed by different groups [26–33] and different cassava genotypes were used; therefore comparisons to our whitefly, SA and JA data likely identify a subset of *PR* gene responses. In each of these interactions, we selected cassava genotypes susceptible to a pathogen/pest and time points similar to those used in the whitefly infestation studies presented here. These data included: the mealybug *P. manihoti* (P40/1; 24 and 72 hpi), the bacteria *X. axonopodis* strain ORST4(*TALE1*_*Xam*_) (MCOL1522; 5 and 7 dpi), the fungus *C. gloeosporioides* (HN; 24 and 72 hpi), and the viruses South African CMV (T200; 12 and 32 dpi) and CBSV (60444; 28 dpi) [26–33]. Expression values from healthy cassava organs were also used (three-month-old TME204) [86]. Time-course expression data used in these studies, as well as our whitefly- and hormone-treatment studies in COL2246, were consolidated to facilitate comparisons. For each time course, we used all treatment time points that were differentially expressed relative to 0 h (|log_2_FC| > 1 and FDR ≤ 5%) and calculated a mean log_2_FC value for each *PR* gene. For the dataset from Muñoz-Bodnar et al. [28], log_2_FC values were calculated as follows: log_2_(FPKM inoculated/FPKM mock). Heatmaps were constructed using the R package ComplexHeatmap [84] and organized along the y-axis according to *PR* phylogenetic clades.

### qRT-PCR

For qRT-PCR, cDNA templates were synthesized using 5 ng of mRNA and the Improm II reverse transcriptase protocol (Promega, Madison, WI). We selected the control gene *UBQ* (Manes.10G122600) based on its low read count variation for all time points in each treatment. qRT-PCR was performed for selected *PR* genes and the *UBQ* control using gene-specific primers in the Bio-rad CFX Connects instrument using iQ SYBR Green Supermix (Bio-rad, Hercules, CA) (Additional file 19). Melting curve analyses were performed at the end of each cycle to confirm the specificity of the PCR product. Relative expression changes were calculated by the comparative Ct method; fold change was calculated as 2^-ΔΔCt^ [87]. Three biological and technical replicates were used for these analyses. Fold-change values are displayed with standard error.

### Pearson correlation analyses

Pearson correlation analyses were performed using the R package ggplot2 [88]. Correlation strength was defined according to Evans [89] as very weak (|0.00–0.19|), weak (|0.20–0.39|), moderate (|0.40–0.59|), strong (|0.60–0.79|), or very strong (|0.80–1.00|). Prior to analyses of biotic stress, outliers were identified using the boxplot rule with a multiplicative constant of 2.0 and removed [90]. Comparisons with fewer than ten DEGs in both treatments (e.g., whiteflies-fungi and whiteflies-mealybugs) did not undergo correlation analysis. No outliers were identified in the SA/JA co-regulation or qRT-PCR vs RNAseq correlation analyses.

## Supplementary information

**Additional file 1: Table S1.** Cassava *PR* gene nomenclature. Founder protein(s) used to identify each *PR* family via BLASTP and HMM queries, a list of cassava *PR* genes and functions, and loci designations in the cassava genome are provided. E-values and % identity are also provided. **Table S2.** *PR* gene clusters in the cassava genome.

**Additional file 2 **Neighbor-joining phylogenetic trees of cassava *PR* families. **Figure S1.** PR-1. **Figure S2.** PR-2. **Figure S3.** PR-3. **Figure S4.** PR-4. **Figure S5.** PR-5. **Figure S6.** PR-6. **Figure S7.** PR-7. **Figure S8.** PR-8. **Figure S9.** PR-9. **Figure S10.** PR-10. **Figure S11.** PR-11. **Figure S12.** PR-14. **Figure S13.** PR-15/16. **Figure S14.** PR-17. Founder (green), cassava (pink), poplar (blue), and rice (black) PR proteins are indicated. Branches with bootstrap values of 50% or higher are shown.

**Additional file 3 ***PR* gene expression after whitefly, SA and JA treatments of cassava. **Table S3.** log_2_FC and FDR values of DEGs identified in whitefly-susceptible genotypes (COL2246, COL1468, 60444, and TME3) after whitefly infestation. **Table S4.** log_2_FC and FDR values for *PR* genes detected in whitefly-susceptible genotypes after whitefly infestation. **Table S5.** Mean RPKM values of *PR* genes during whitefly infestation (0–22 dpi) of four whitefly-susceptible genotypes. **Table S6.** Read counts of *PR* genes during whitefly infestation (0–22 dpi) of four whitefly-susceptible genotypes. **Table S7.** log_2_FC and FDR values of DEGs in COL2246 after SA treatment. **Table S8.** log_2_FC and FDR values for *PR* genes detected in COL2246 after SA treatment. **Table S9.** Mean RPKM values for *PR* genes after SA treatment (0–24 h) of COL2246. **Table S10.** Read counts of *PR* genes after SA treatments (0–24 h) of COL2246**. Table S11.** log_2_FC and FDR values of DEGs in COL2246 after JA treatment. **Table S12**. log_2_FC and FDR values for *PR* genes detected in COL2246 after JA treatment. **Table S13.** Mean RPKM values for *PR* genes after JA treatment (0–24 h) of COL2246. **Table S14.** Read counts for *PR* genes after JA treatment (0–24 h) of COL2246.

**Additional file 4 Table S15.** Expression profile clusters for *PR* responses to whitefly feeding. **Table S16.** Expression profile clusters for *PR* responses to whitefly, SA and JA.

**Additional file 5 **Venn diagrams comparing cluster 9 downregulated *PR* genes among four whitefly-susceptible cassava genotypes during whitefly infestation. (a) Comparison of cluster 9 DEGs in COL2246, COL1468, 60444, and TME3 during whitefly infestation. (b) Comparison of COL2246 cluster 9 DEGs at 1–22 dpi. (c) Comparison of COL1468 cluster 9 DEGs at 1–22 dpi. (d) Comparison of 60444 cluster 9 DEGs at 1–22 dpi. (e) Comparison of TME3 cluster 9 DEGs at 1–22 dpi.

**Additional file 6 **Venn diagrams comparing cluster 1 upregulated *PR* genes among four whitefly-susceptible cassava genotypes during whitefly infestation. (a) Comparison of cluster 1 DEGs in COL2246, COL1468, 60444, and TME3 during whitefly infestation. (b) Comparison of COL2246 cluster 1 DEGs at 1–22 dpi. (c) Comparison of COL1468 cluster 1 DEGs at 1–22 dpi. (d) Comparison of 60444 cluster 1 DEGs at 1–22 dpi. (e) Comparison of TME3 cluster 1 DEGs at 1–22 dpi.

**Additional file 7.** Mean log_2_FC of SA and/or JA DEGs.

**Additional file 8.** Whitefly-responsive DEGs: Temporal expression programs and hormone-response clusters.

**Additional file 9 ***PR* gene expression values (log_2_FC) during biotic stress. This table compiles DEGs identified in response to: SA, JA and whitefly (this study); *Xanthomonas* (bacteria) [27, 28]; *C. gloeosporioides* (fungi) [29]; and the viruses South African CMV [30] and CBSV [31–33].

**Additional file 10 ***PR-1, PR-2* and *PR-3* family member phylogenies and consolidated gene expression heatmaps are displayed. Genes within a clade are designated by a letter and color bars in the circular phylogenetic trees and heatmaps. Information about physical clustering and cassava-specific expansions are provided beside the heatmaps, which provide gene expression changes during biotic stresses or hormone treatments (SA and JA) and in shoots and storage roots. Recent *PR* family expansions are shown in red in the circular trees and expansion column; other genes (light grey) in the expansion column are not part of cassava-specific *PR* family expansions (see Methods). Genes belonging to the same physical cluster are denoted with the same color in the cluster column; genes that do not belong to a cluster are in light grey. Genes displayed as dark grey do not have an assigned chromosomal position in the cassava genome ver. 6. **Figure S15.** *PR-1.* **Figure S16.** *PR-2.* **Figure S17.** *PR-3.*

**Additional file 11 ***PR-4, PR-5* and *PR-6* family member phylogenies and consolidated gene expression heatmaps are displayed. The *PR-6* family phylogenetic tree is not displayed due to its small size. **Figure S18.** *PR-4.* **Figure S19.** *PR-5.* **Figure S20.** *PR-6.*

**Additional file 12 ***PR-7* and *PR-8* family member phylogenies and consolidated gene expression heatmaps are displayed. **Figure S21.** *PR-7.* **Figure S22.** *PR-8.*

**Additional file 13 ***PR-9, PR-10* and *PR-11* family member phylogenies and consolidated gene expression heatmaps are displayed. **Figure S23.** *PR-9.* **Figure S24.** *PR-10.* **Figure S25.** *PR-11.*

**Additional file 14 ***PR-14, PR-15/16* and *PR-17* family member phylogenies and consolidated gene expression heatmaps are displayed. **Figure S26.** *PR-14.* **Figure S27.** *PR-15/16.* **Figure S28.** *PR-17.*

**Additional file 15 **Hormone regulation of stress-responsive *PR* genes. Numbers and percentages of stress-regulated genes belonging to each hormone-expression program are provided. **Table S17.** Hormone regulation of whitefly-responsive *PR* genes. **Table S18.** Hormone regulation of mealybug-responsive *PR* genes. **Table S19.** Hormone regulation of bacteria-responsive *PR* genes. **Table S20.** Hormone regulation of fungi-responsive *PR* genes. **Table S21.** Hormone regulation of CMV-responsive *PR* genes. **Table S22.** Hormone regulation of CBSV-responsive *PR* genes.

**Additional file 16 Table S23.** *PR* genes associated with correlations between whitefly and bacteria and/or CBSV responses. **Table S24.** *PR* genes associated with correlation between whitefly and CMV responses.

**Additional file 17 ***PR* gene expression in TME204 shoots, roots and embryonic callus. Loci (cassava genome v6), *PR* gene names and expression values from Wilson et al. [86] are provided. These data are used in Additional files 12, 13, 14, 15 and 16.

**Additional file 18.** Pearson correlations of count values obtained for three biological replicates for all whitefly infestation and hormone treatments. (a) Correlations for SA and JA treatments (0, 0.5, 1, 2, 4, 8, 12, 24 h). (b) Correlations for whitefly infestations (0, 1, 7, 14, and 22 d) for COL2246, COL1468, 60444, and TME3.

**Additional file 19.** qRT-PCR primers.

## Data Availability

All data generated during this study are included in this published article and its supporting information files. Read counts and analyzed data are provided.

## Abbreviations

CBSV: Cassava Brown Streak Virus
CMV: South African Cassava Mosaic Virus
DEGs: Differentially expressed genes
HMM: Hidden Markov Model
JA: Jasmonic acid
PAMP: Pathogen-associated molecular patterns
PR: Pathogenesis-related
qRT-PCR: quantitative real time polymerase chain reaction
SA: Salicylic acid
