# Supplementary figures and images for: Genome-wide analyses of cassava Pathogenesis-related (PR) gene families reveal core transcriptome responses to whitefly infestation, salicylic acid and jasmonic acid
Source: BMC Genomics. 2020 Jan 29;21:93. doi: 10.1186/s12864-019-6443-1 (PMC6990599; doi:10.1186/s12864-019-6443-1)

# PR-1

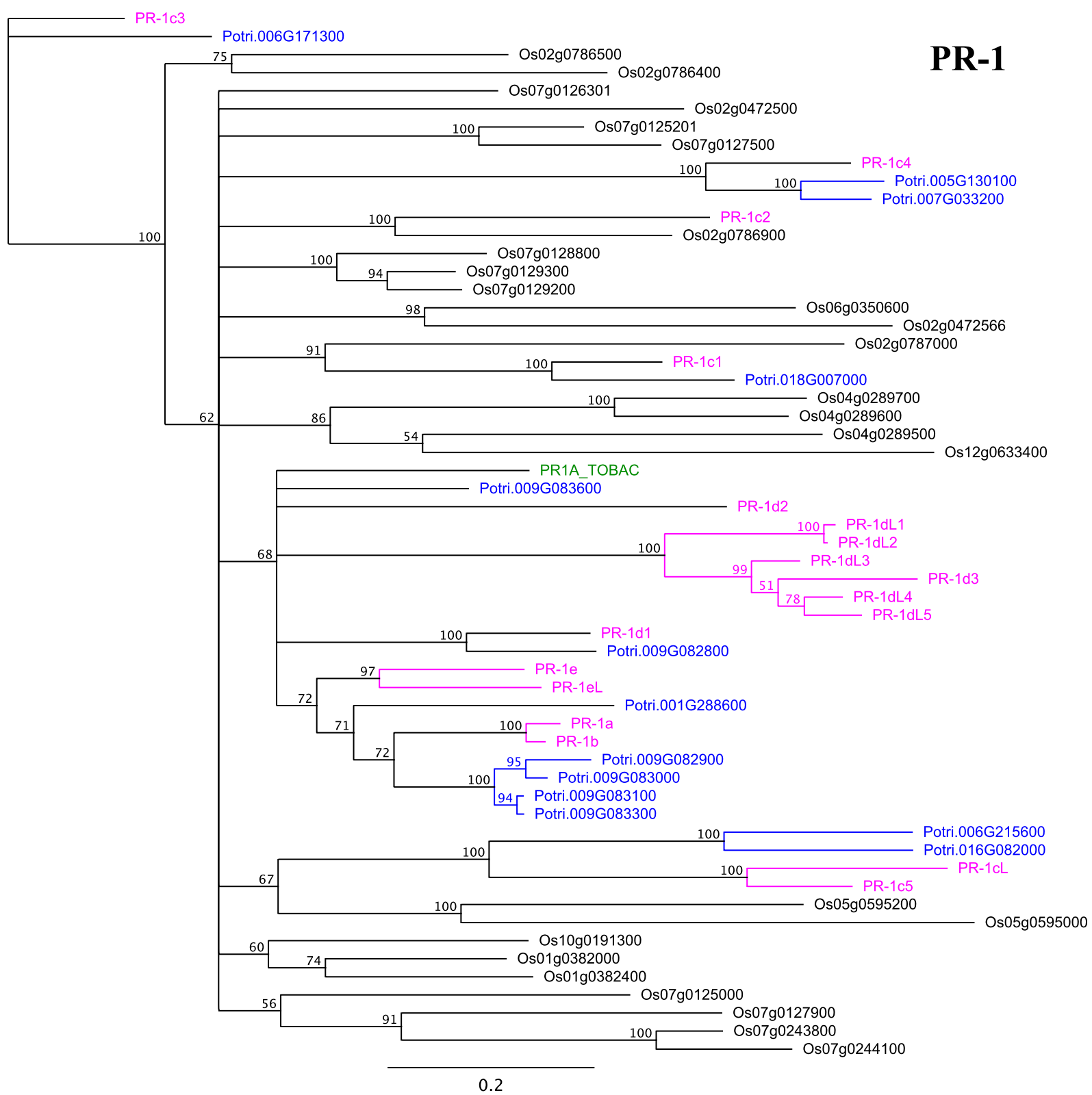

## PR-2

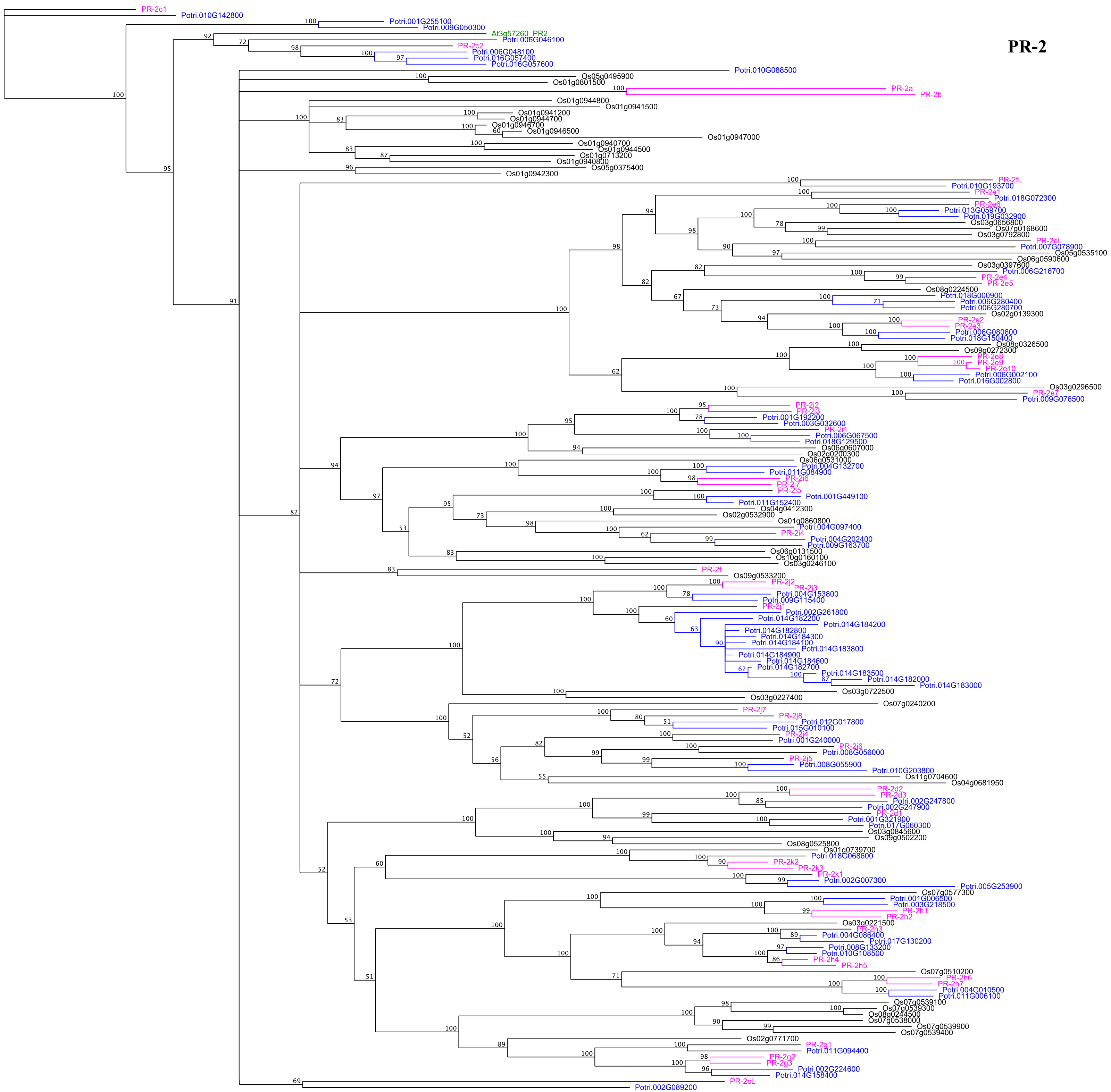

0.2

# PR-3

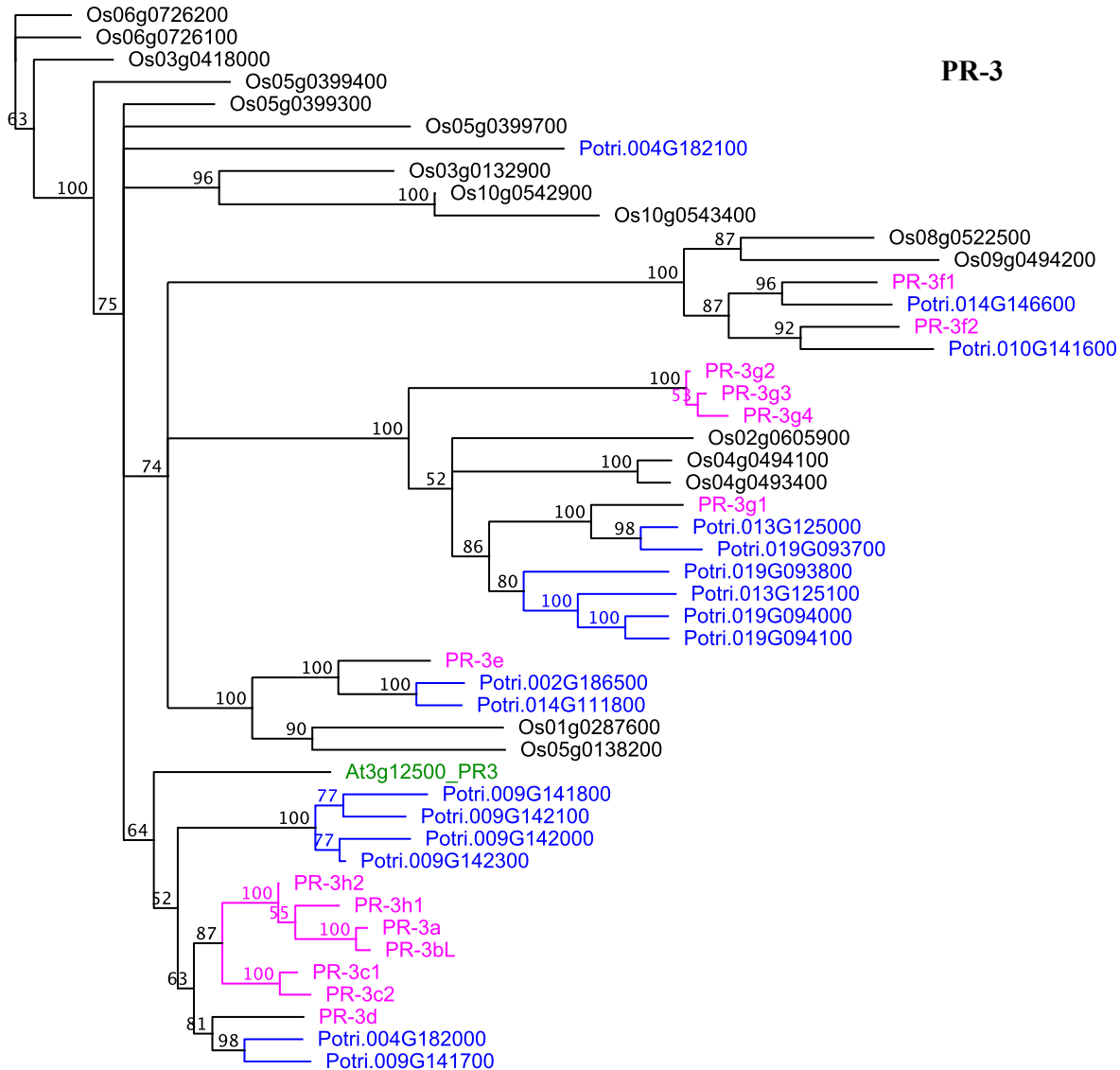

# PR-4

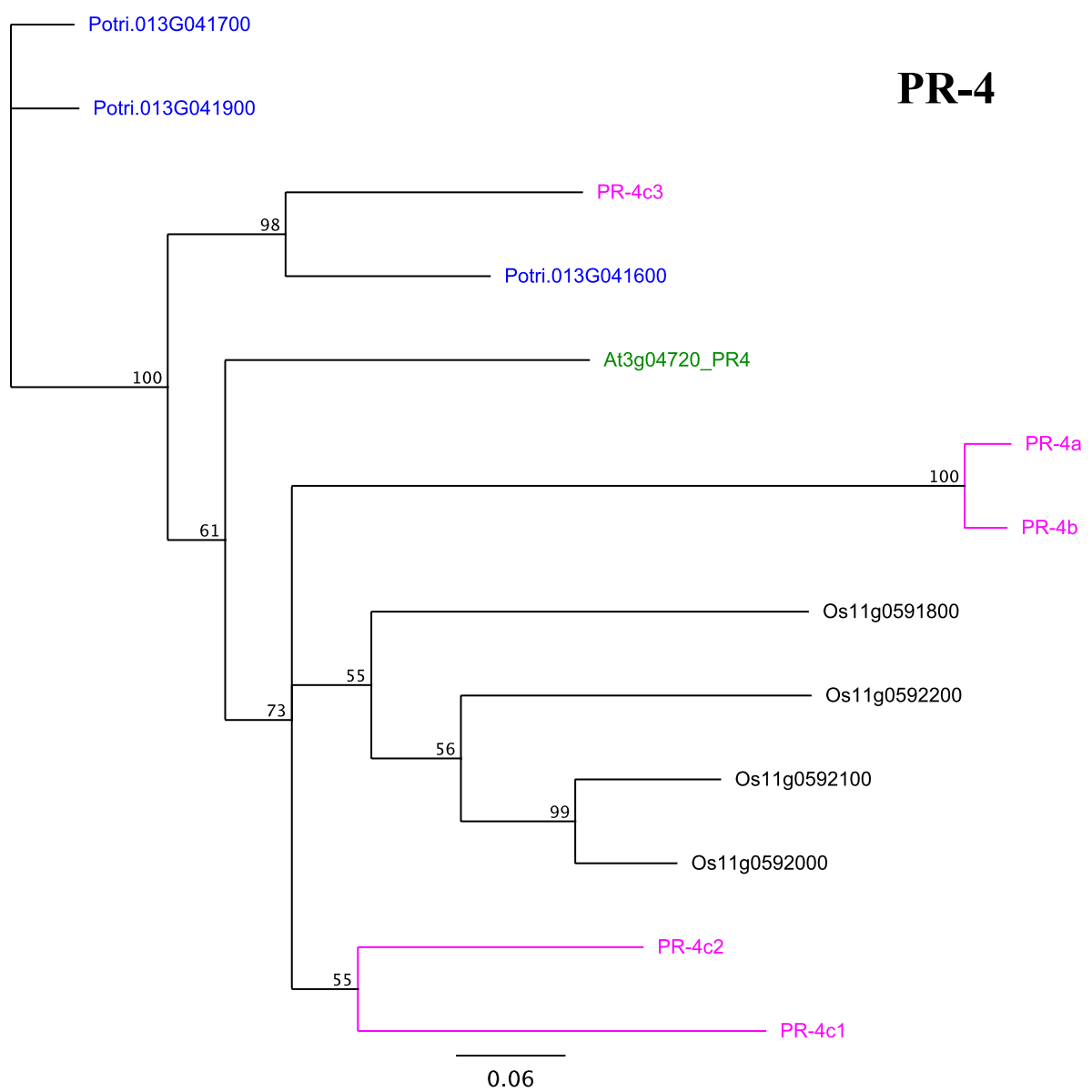

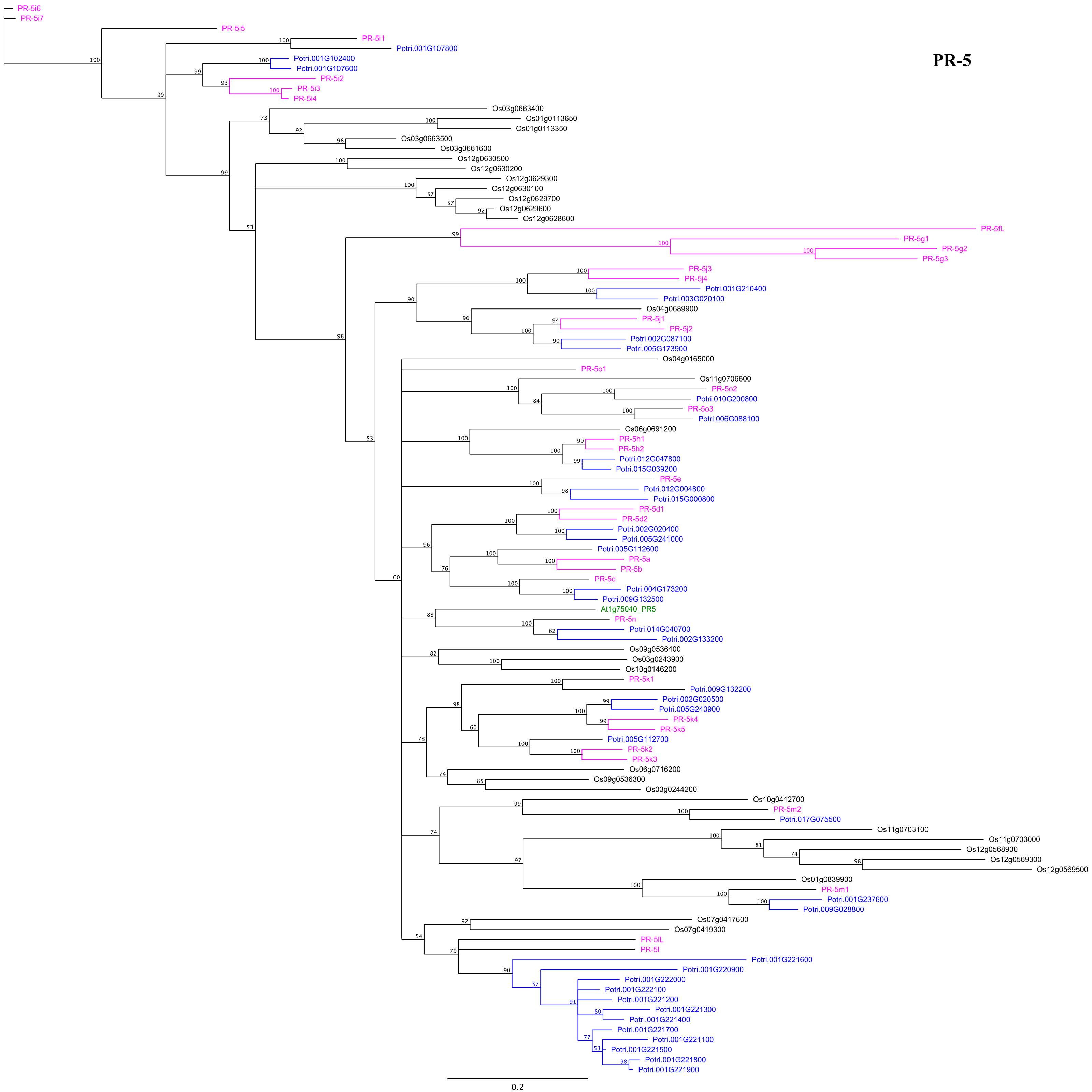

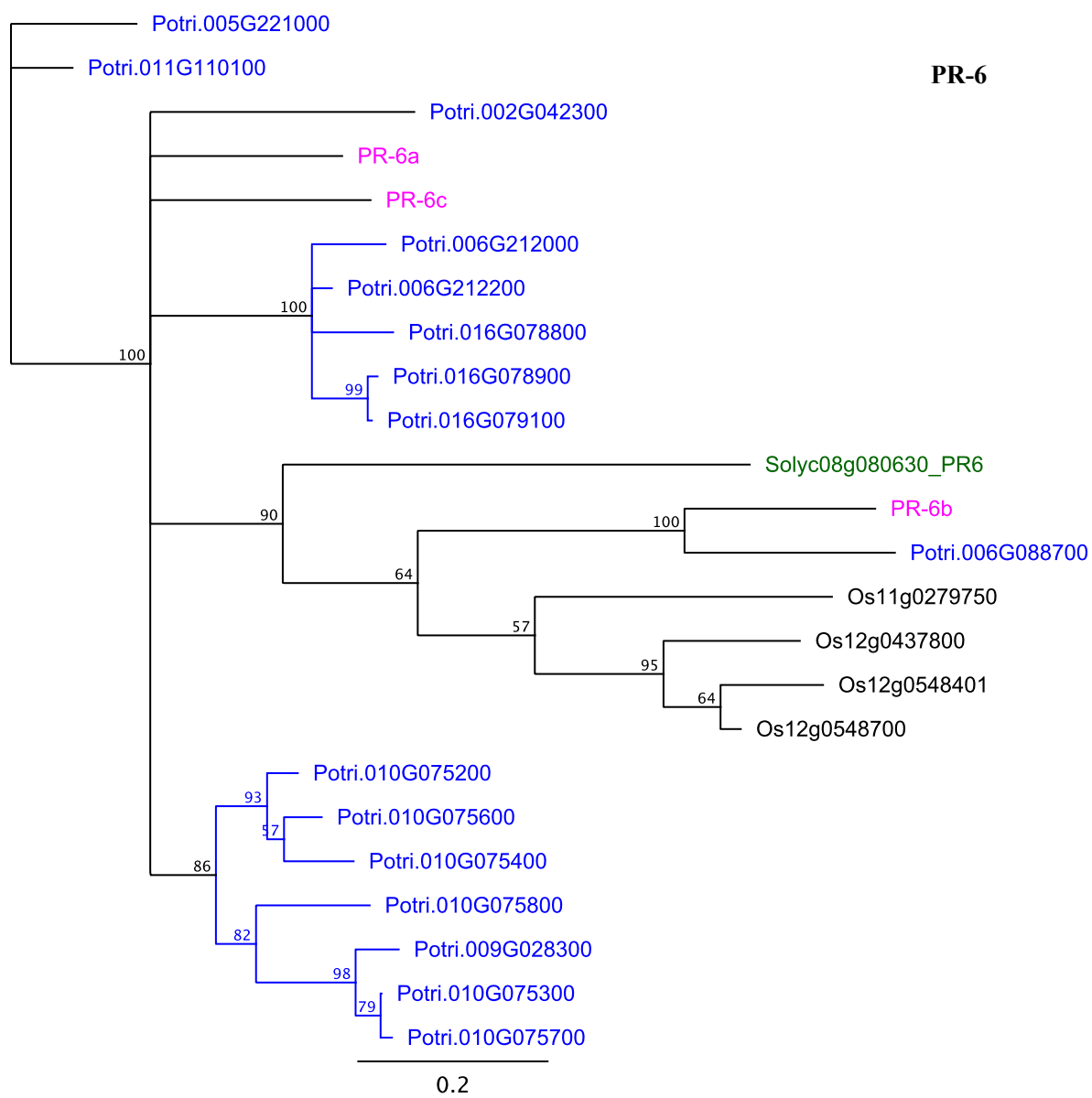

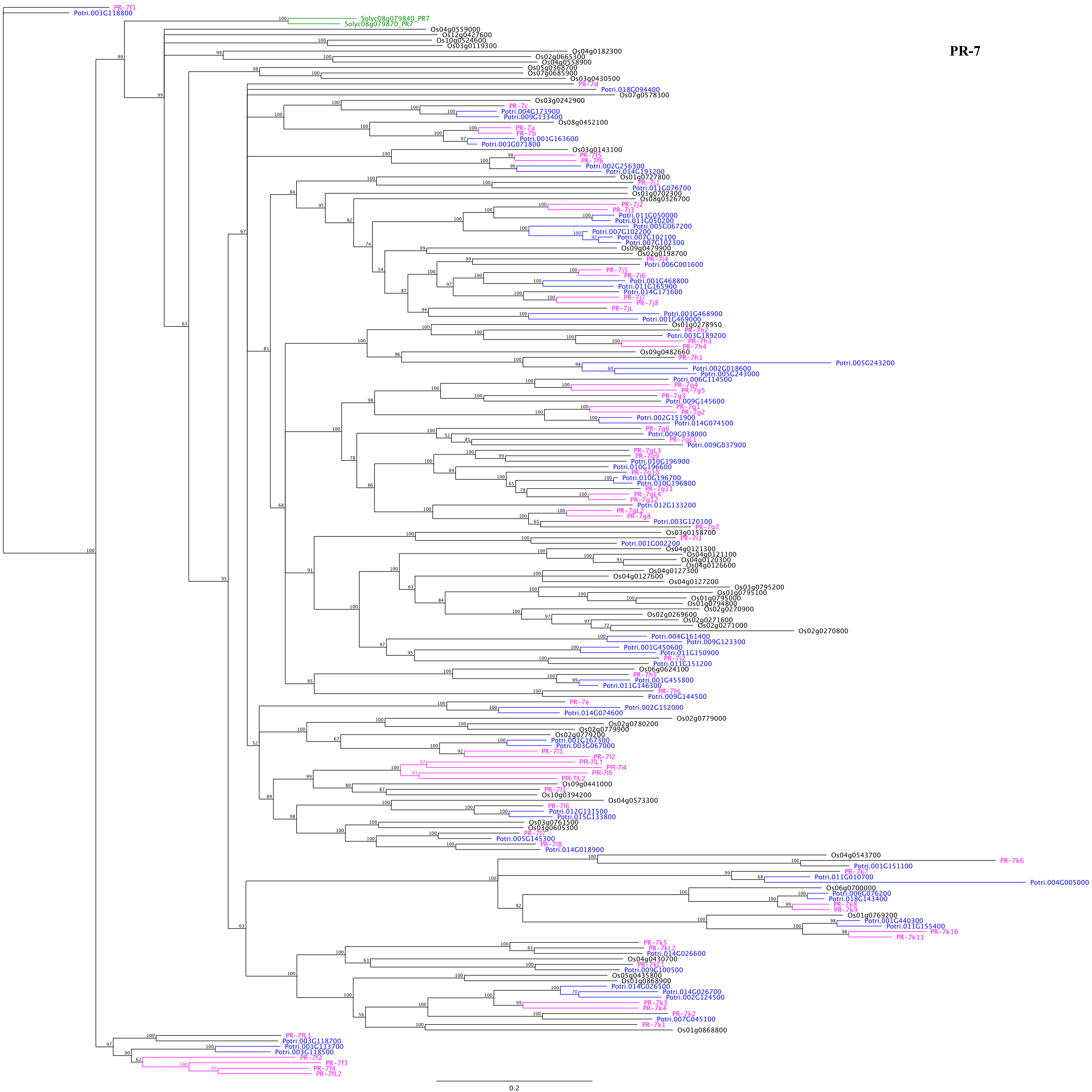

PR-7

0.2

# PR-8

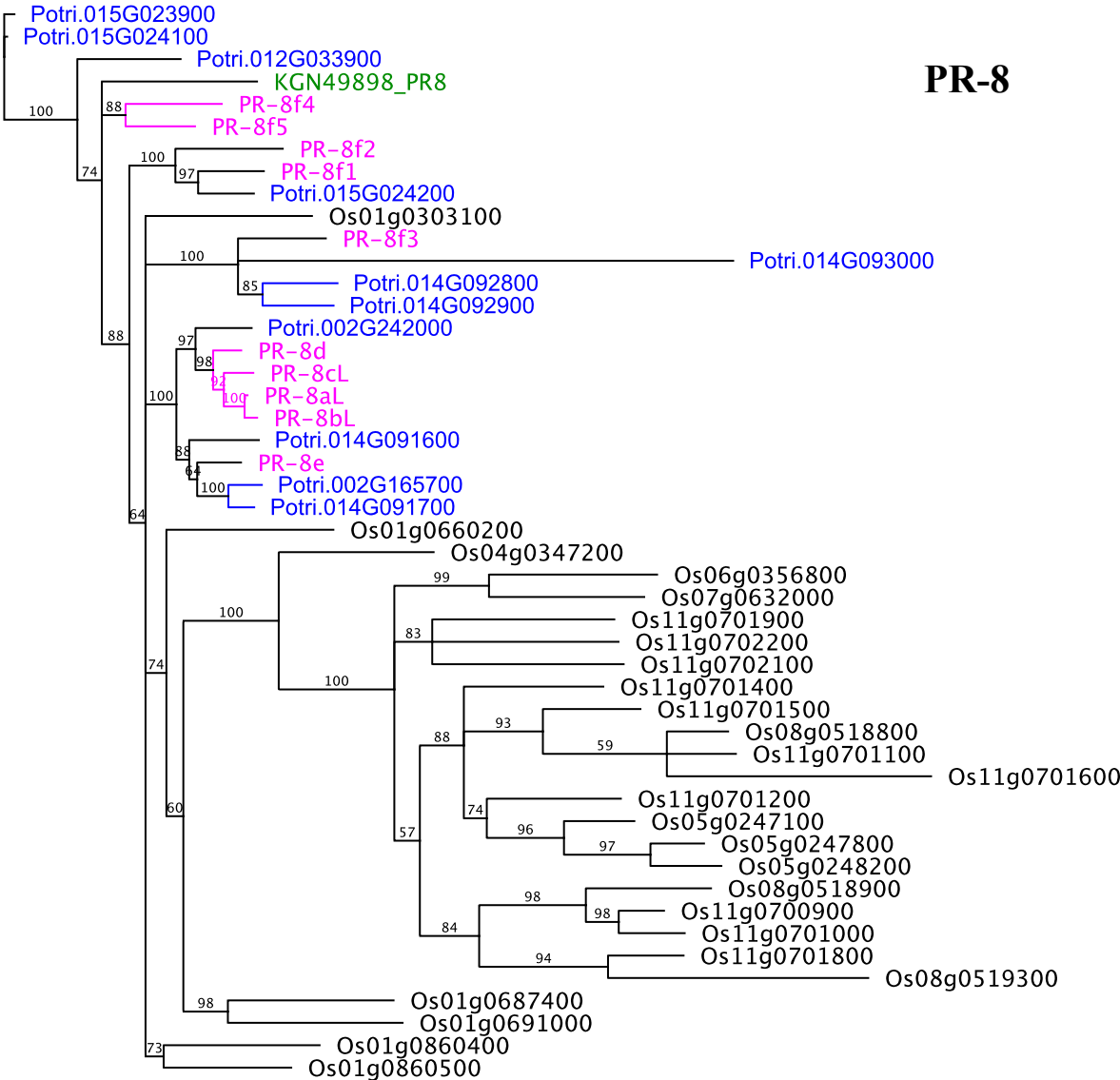

PR-9

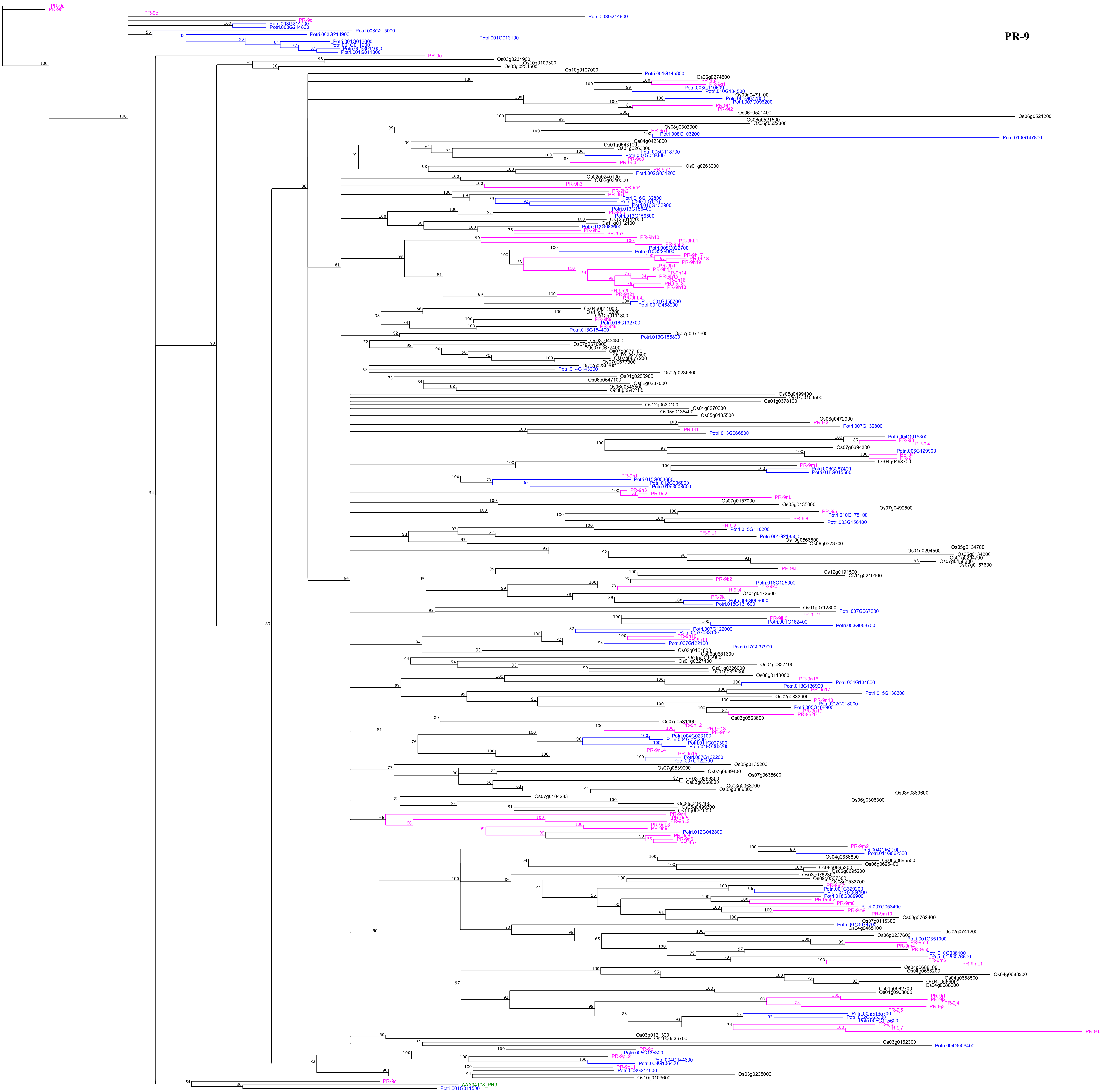

# PR-10

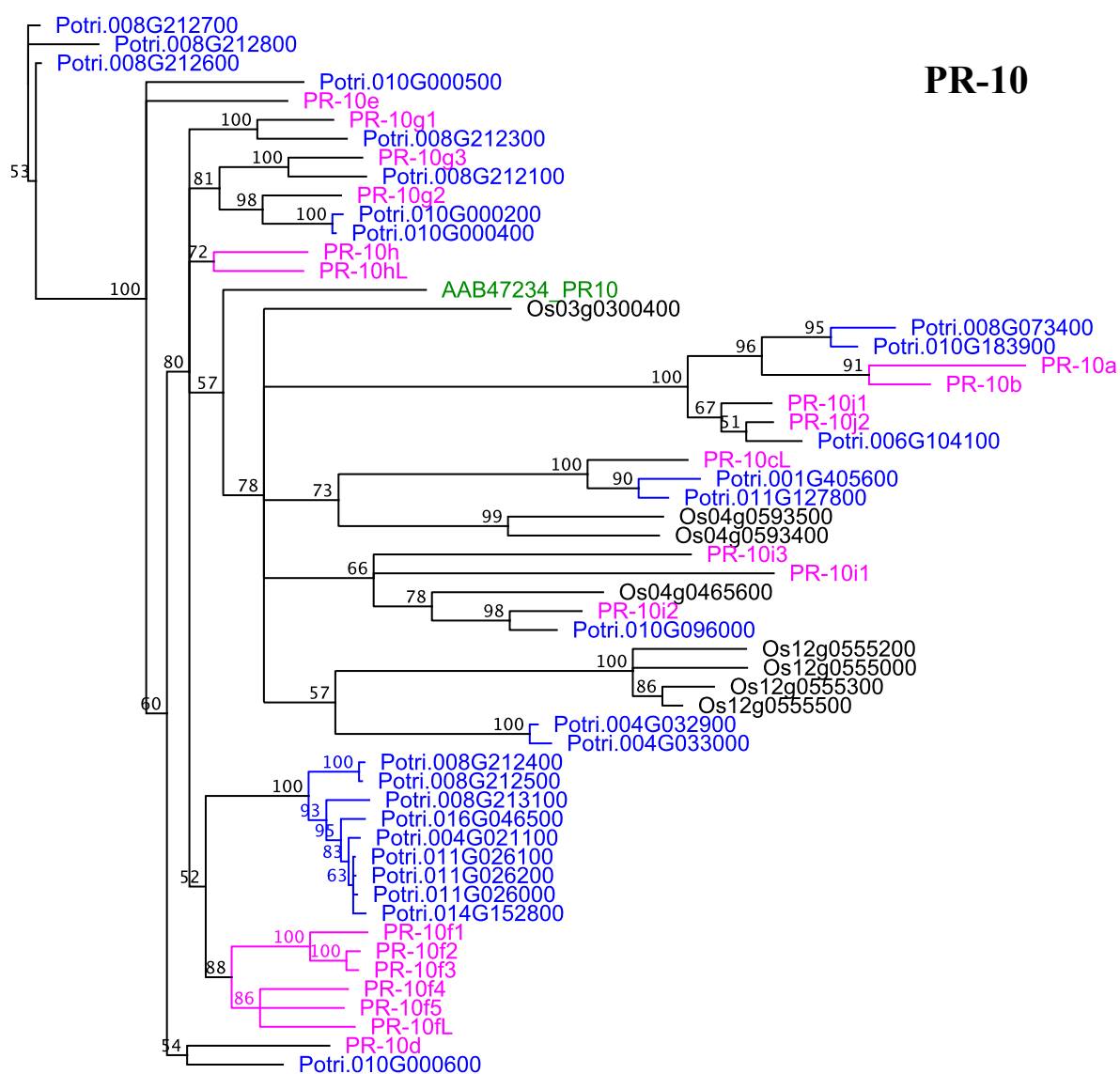

# PR-11

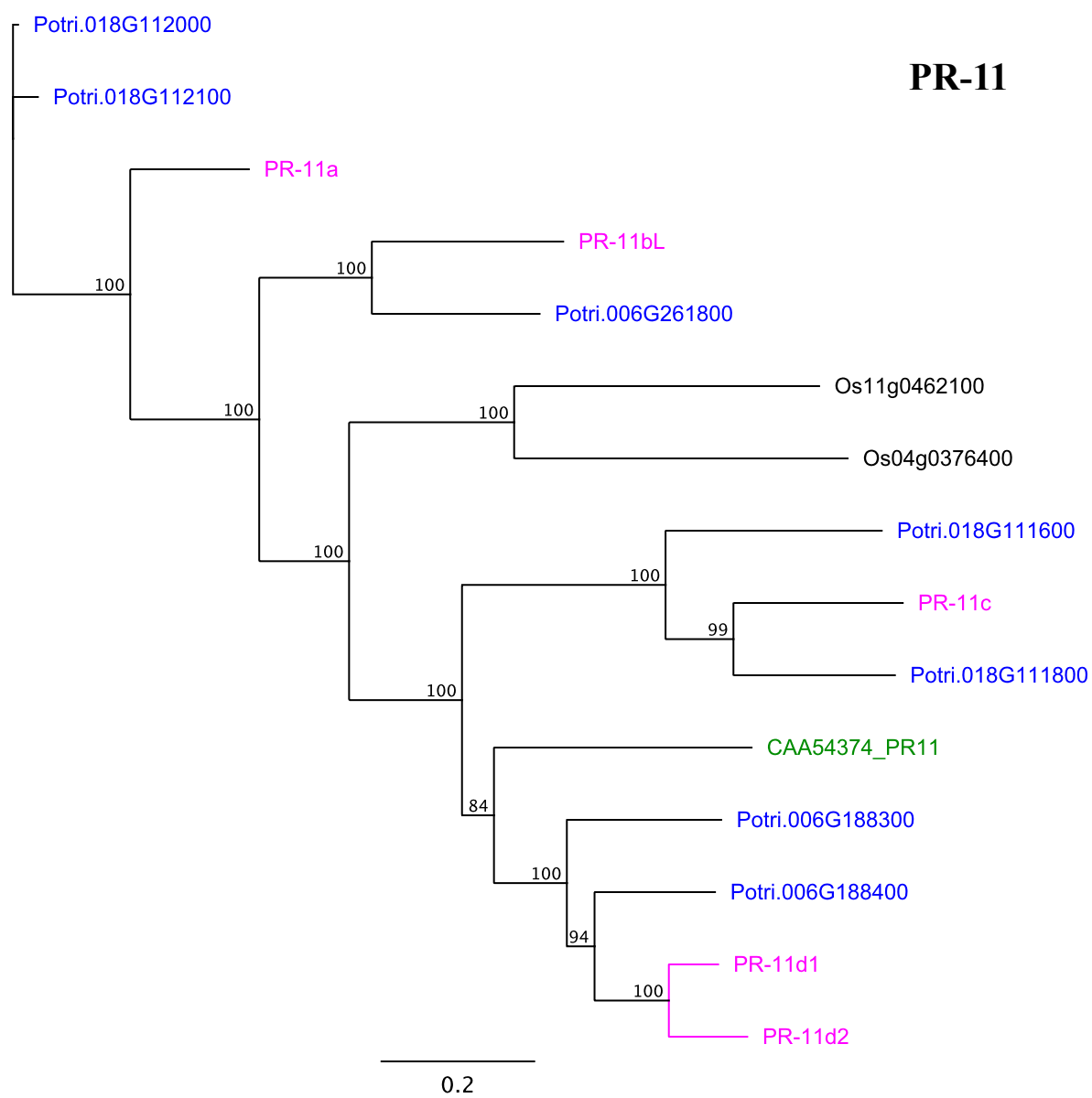

PR-14f  
Potri.012G139700

PR-14

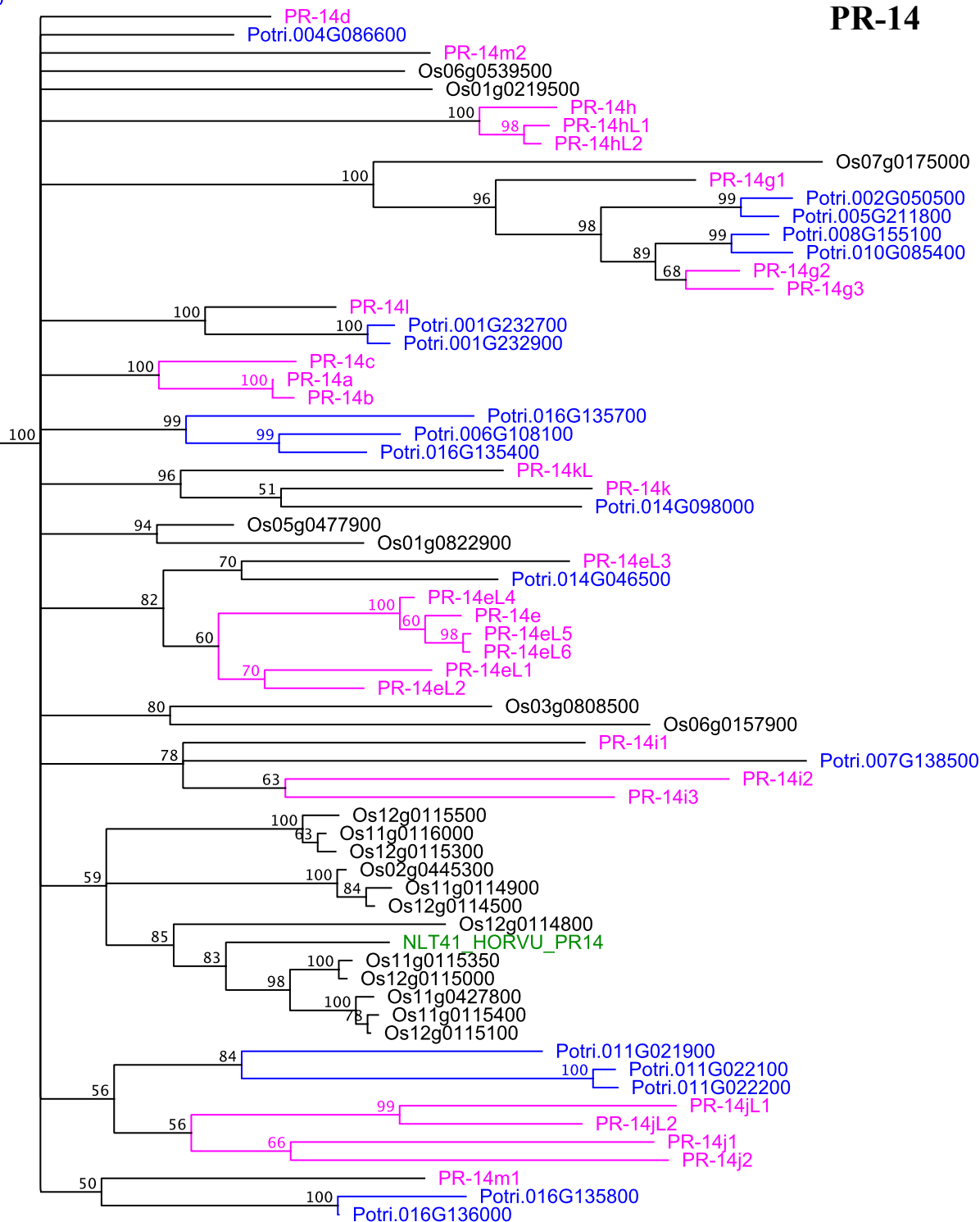

0.2

PR-15\_16e1  
PR-15\_16eL1

Os03g0336100

PR-15/16

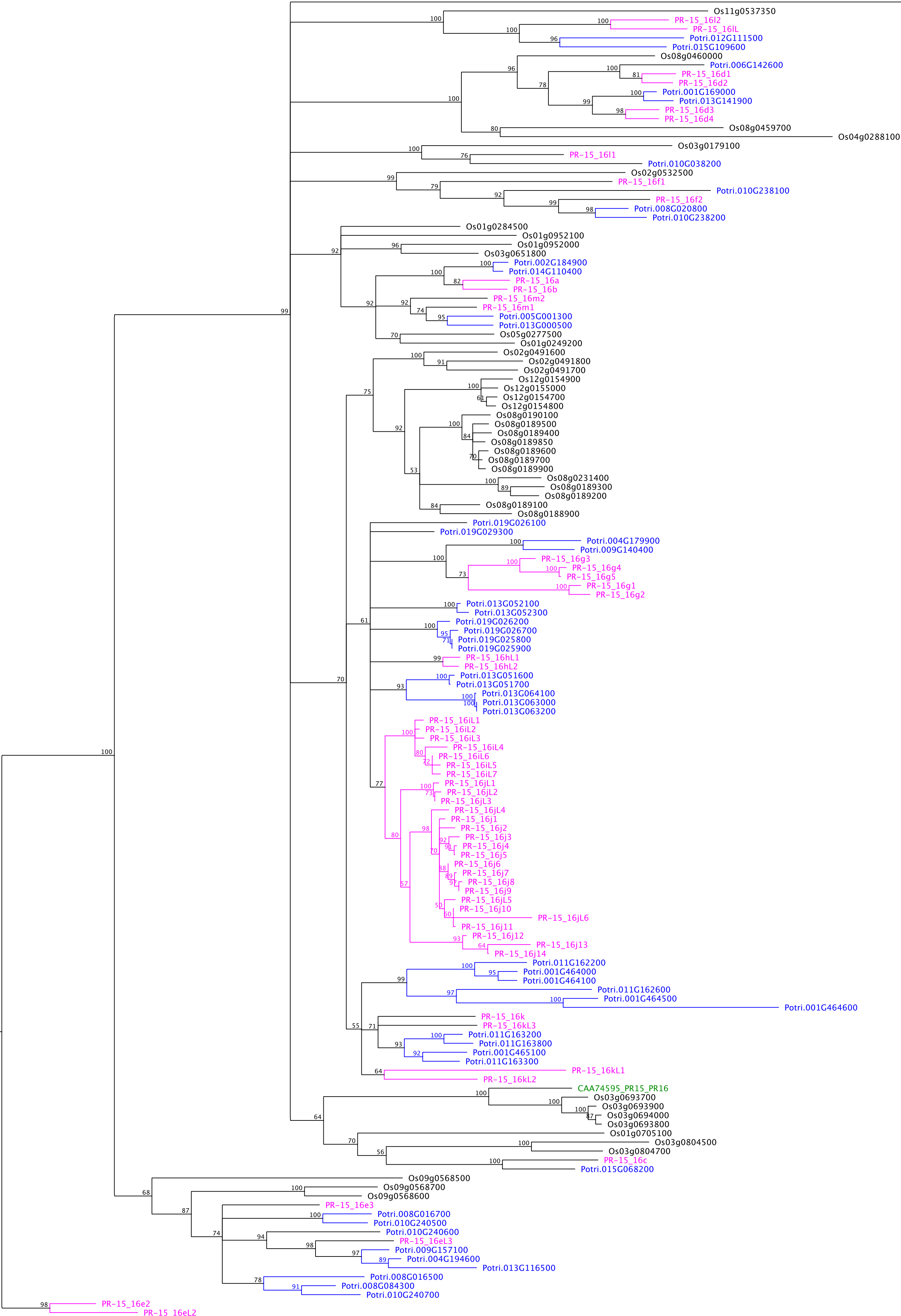

# PR-17

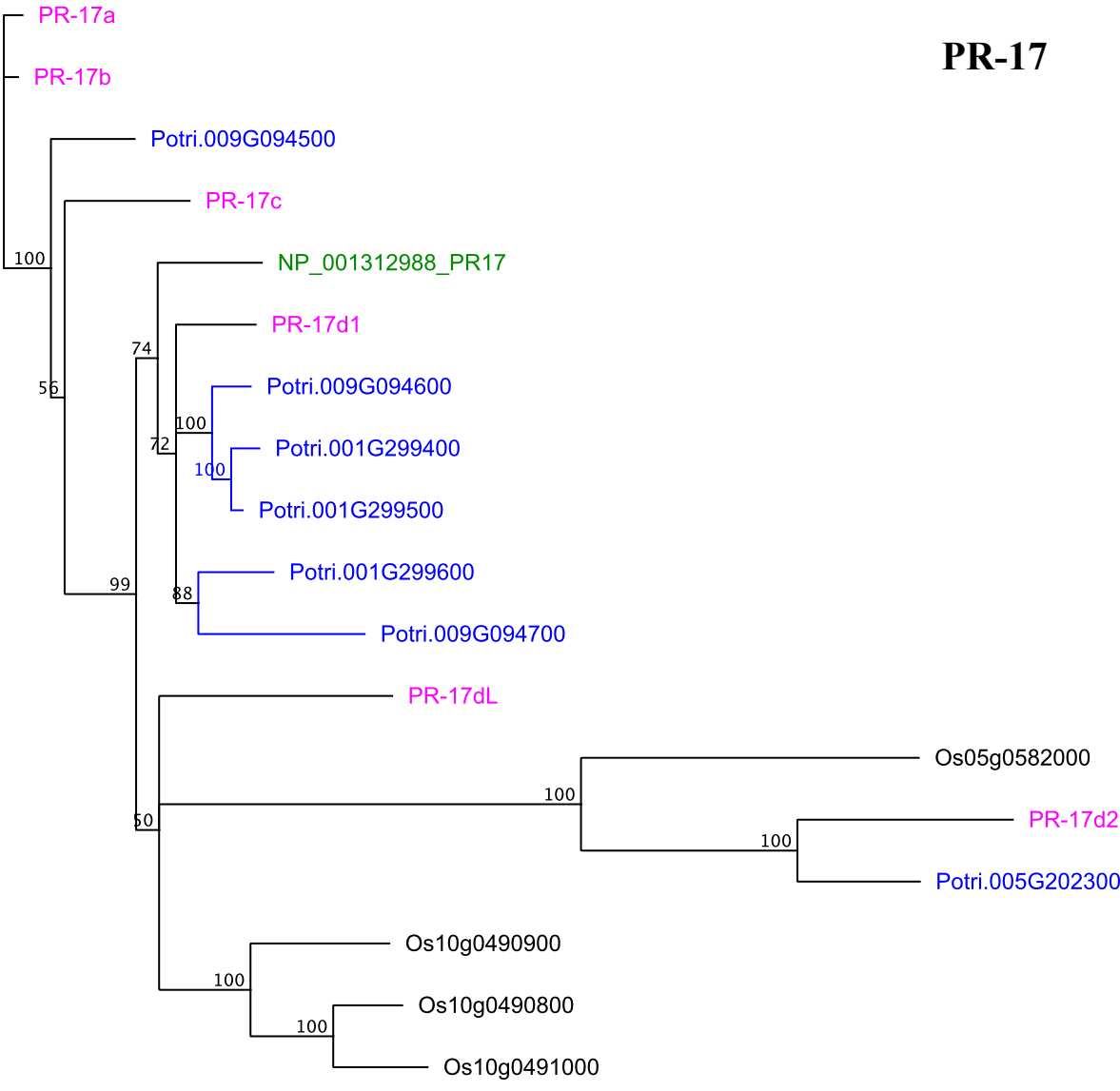

0.3

Supplement: Supplementary file 2 — Additional file 2 Neighbor-joining phylogenetic trees of cassava PR families. Figure S1. PR-1. Figure S2. PR-2. Figure S3. PR-3. Figure S4. PR-4. Figure S5. PR-5. Figure S6. PR-6. Figure S7. PR-7. Figure S8. PR-8. Figure S9. PR-9. Figure S10. PR-10. Figure S11. PR-11. Figure S12. PR-14. Figure S13. PR-15/16. Figure S14. PR-17. Founder (green), cassava (pink), poplar (blue), and rice (black) PR proteins are indicated. Branches with bootstrap values of 50% or higher are shown. [file 12864_2019_6443_MOESM2_ESM.pdf]

**a**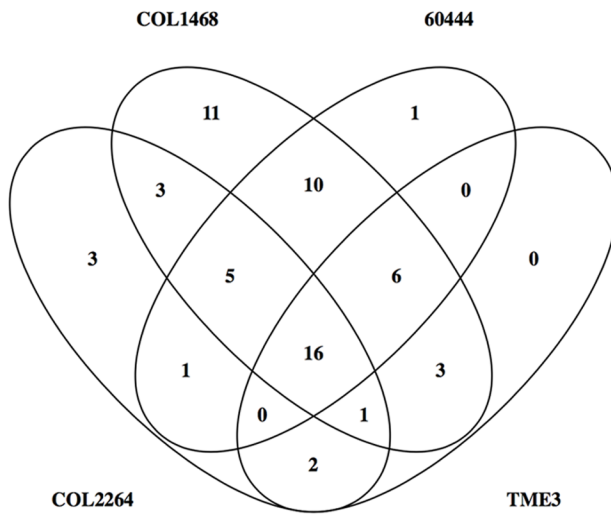**b**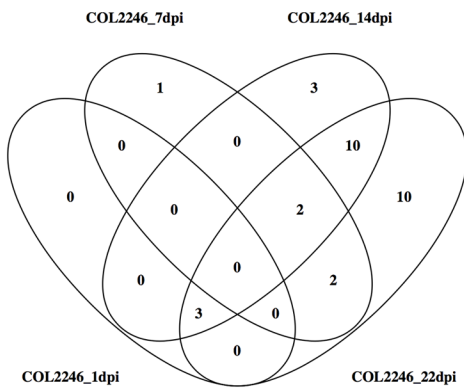**c**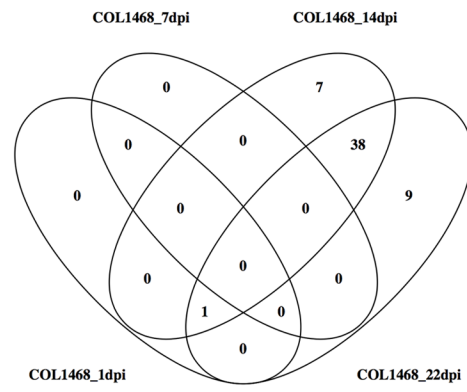**d**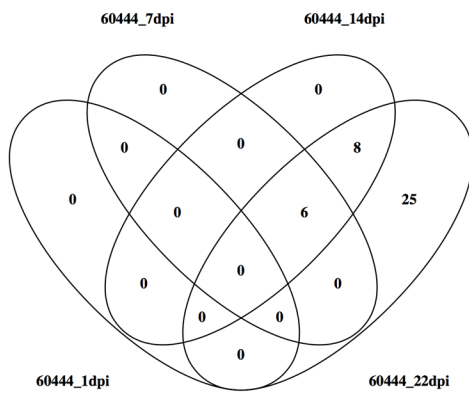**e**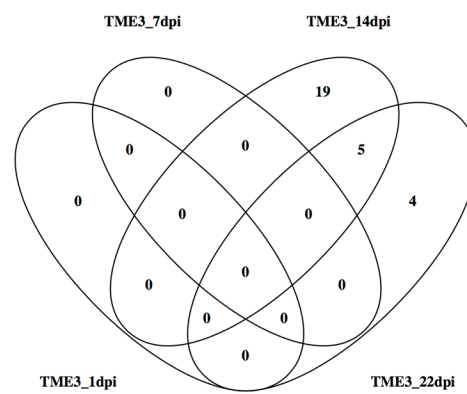

Supplement: Supplementary file 5 — Additional file 5 Venn diagrams comparing cluster 9 downregulated PR genes among four whitefly-susceptible cassava genotypes during whitefly infestation. (a) Comparison of cluster 9 DEGs in COL2246, COL1468, 60444, and TME3 during whitefly infestation. (b) Comparison of COL2246 cluster 9 DEGs at 1–22 dpi. (c) Comparison of COL1468 cluster 9 DEGs at 1–22 dpi. (d) Comparison of 60444 cluster 9 DEGs at 1–22 dpi. (e) Comparison of TME3 cluster 9 DEGs at 1–22 dpi. [file 12864_2019_6443_MOESM5_ESM.pdf]

**a**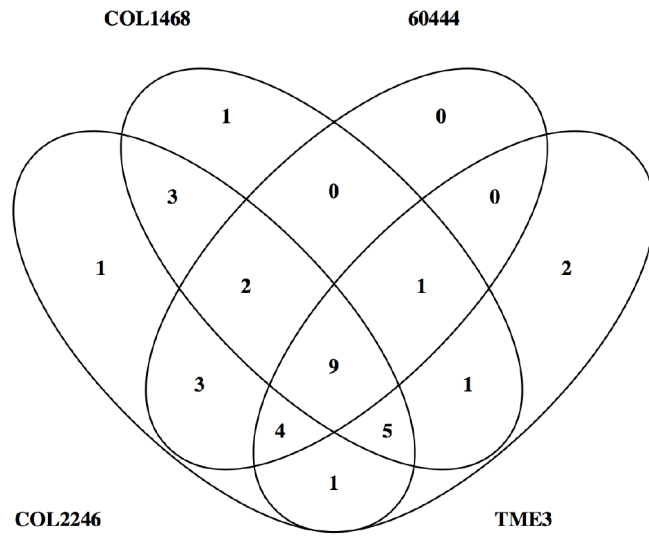**b**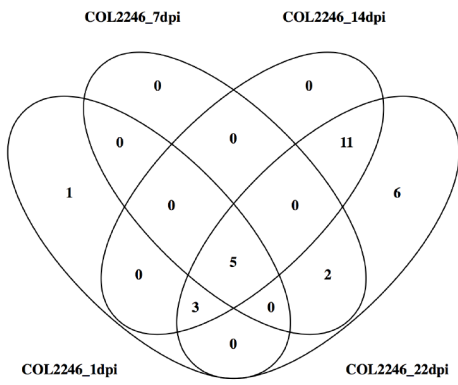**c**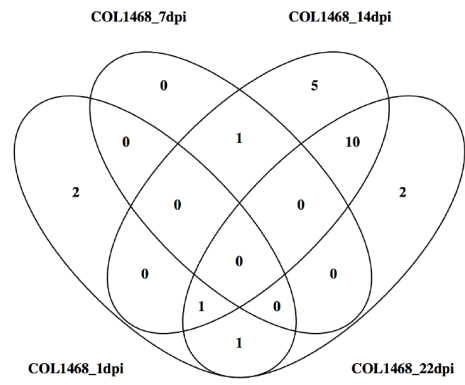**d**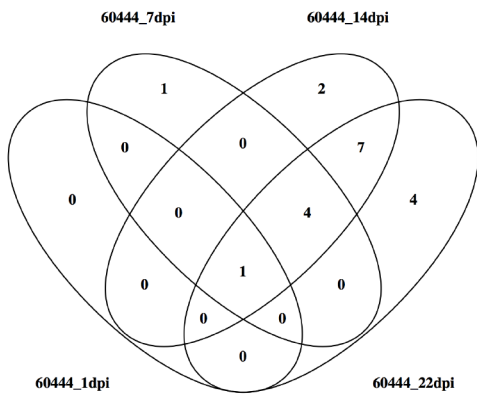**e**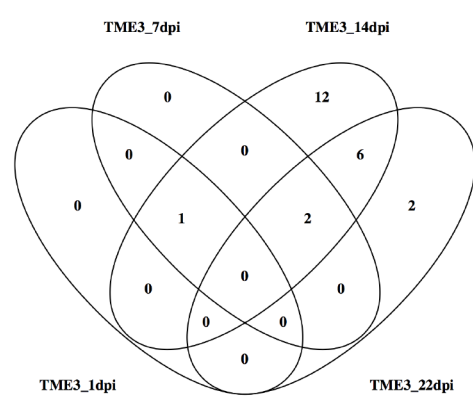

Supplement: Supplementary file 6 — Additional file 6 Venn diagrams comparing cluster 1 upregulated PR genes among four whitefly-susceptible cassava genotypes during whitefly infestation. (a) Comparison of cluster 1 DEGs in COL2246, COL1468, 60444, and TME3 during whitefly infestation. (b) Comparison of COL2246 cluster 1 DEGs at 1–22 dpi. (c) Comparison of COL1468 cluster 1 DEGs at 1–22 dpi. (d) Comparison of 60444 cluster 1 DEGs at 1–22 dpi. (e) Comparison of TME3 cluster 1 DEGs at 1–22 dpi. [file 12864_2019_6443_MOESM6_ESM.pdf]

# PR-7

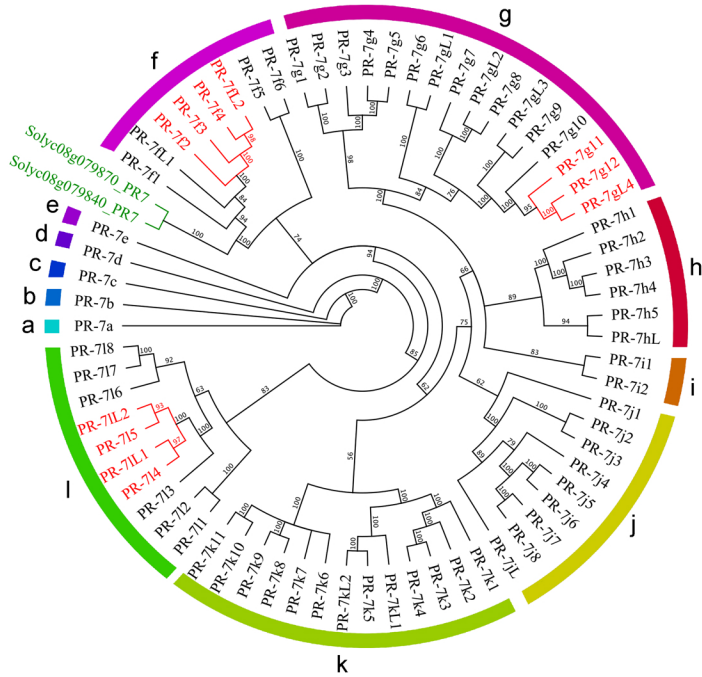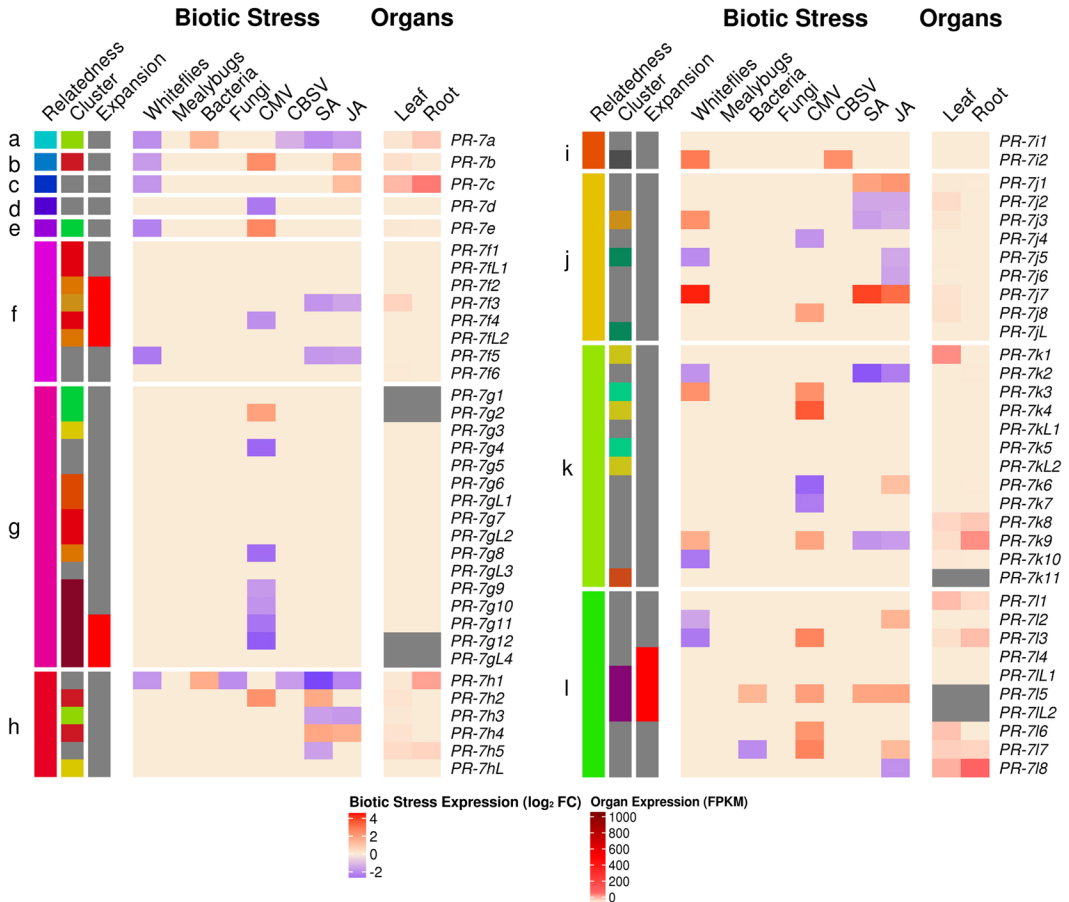

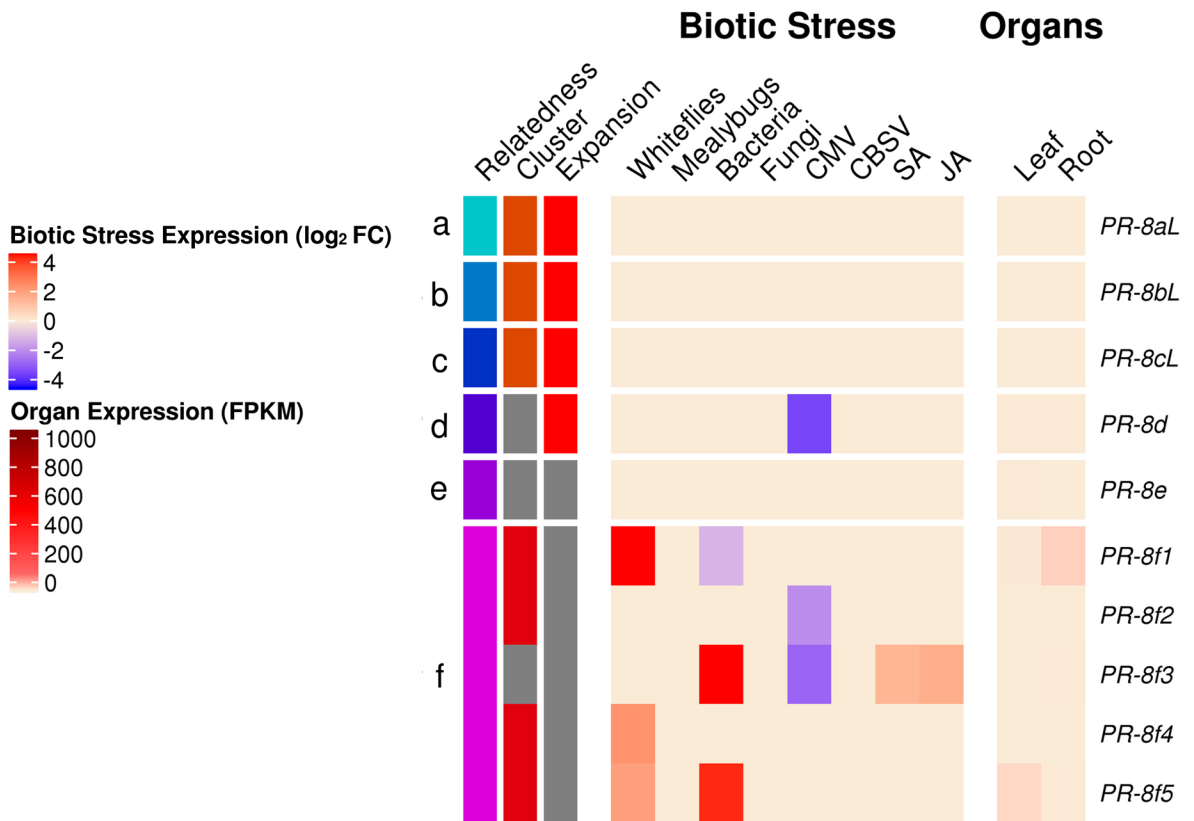

Supplement: Supplementary file 12 — Additional file 12 PR-7 and PR-8 family member phylogenies and consolidated gene expression heatmaps are displayed. Figure S21. PR-7. Figure S22. PR-8. [file 12864_2019_6443_MOESM12_ESM.pdf]

**a**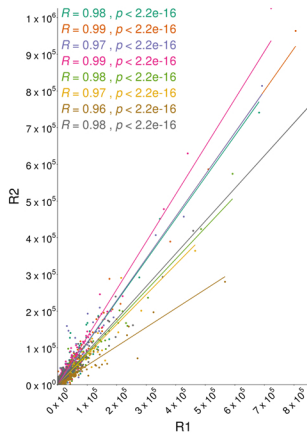**SA**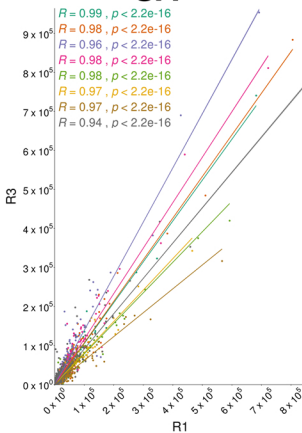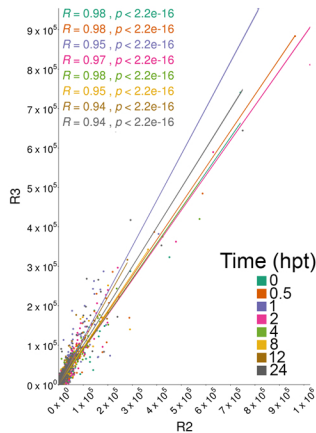

Time (hpt)

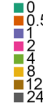**JA**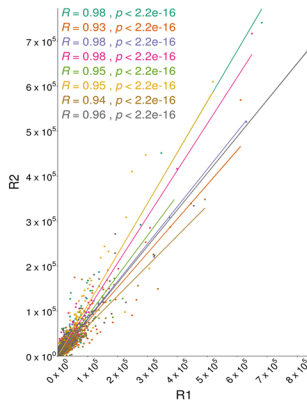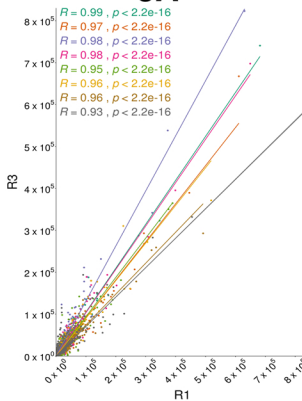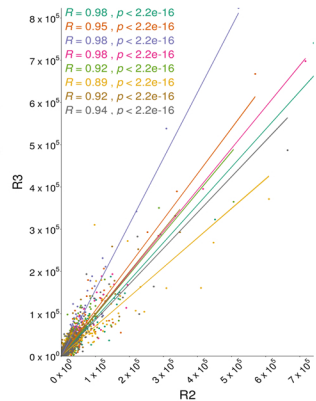

**b**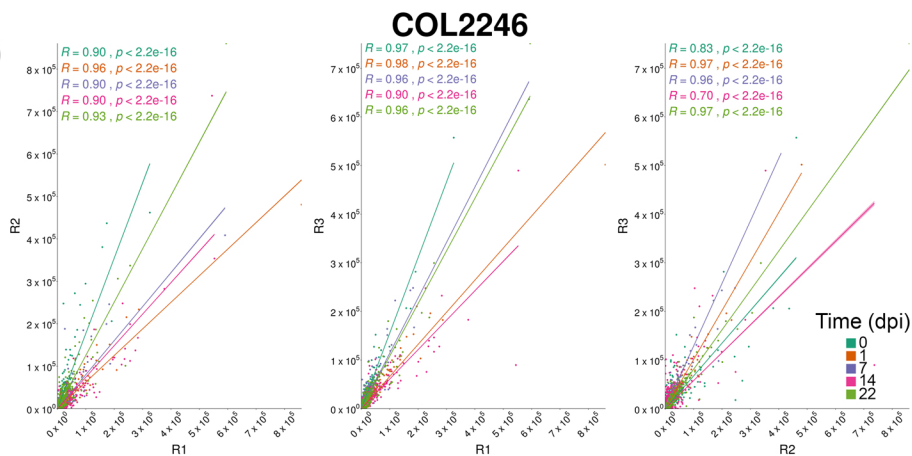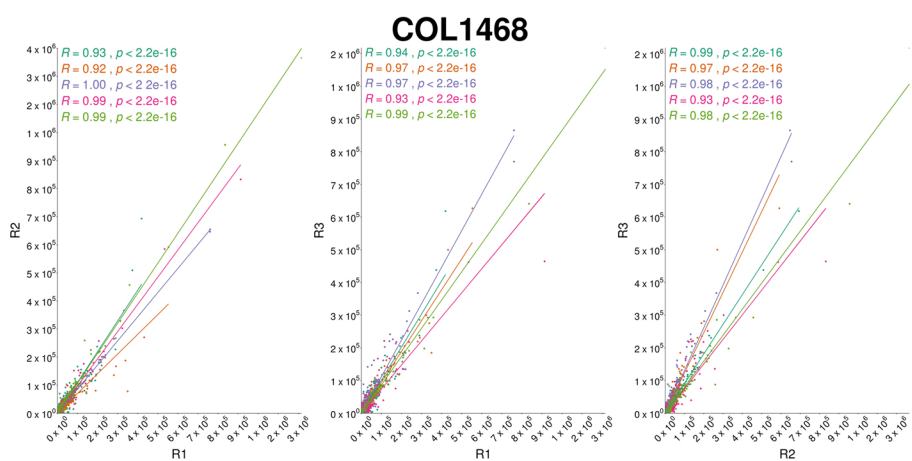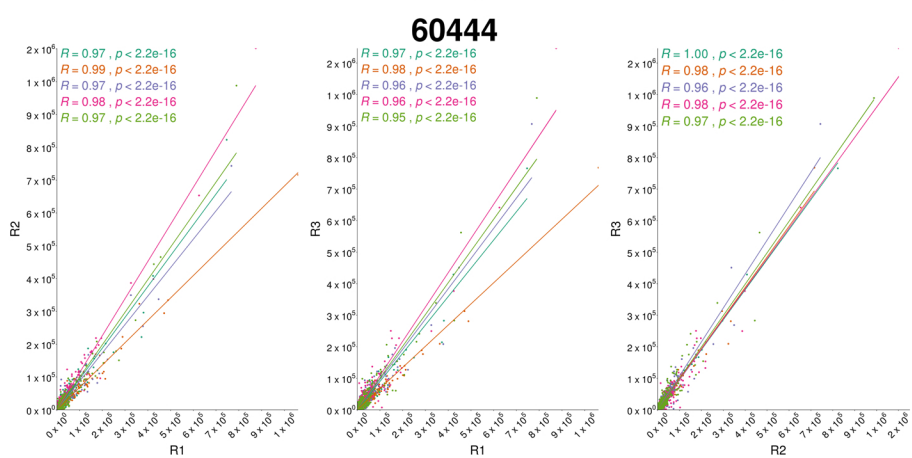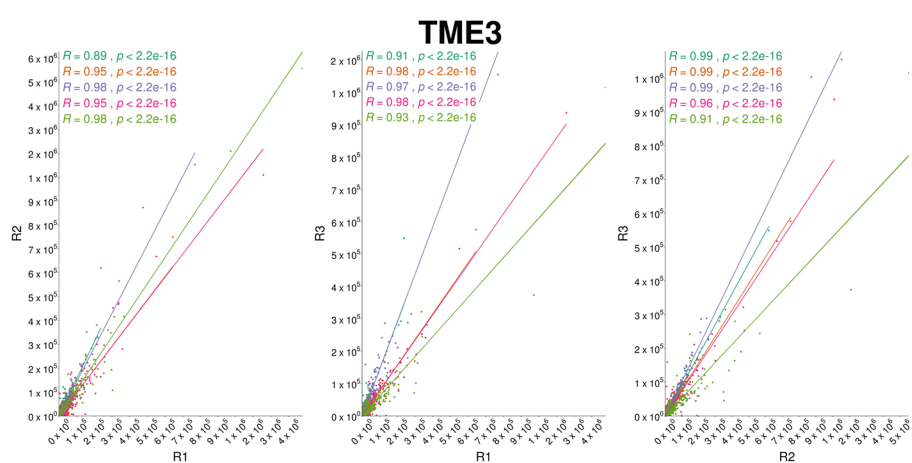

Supplement: Supplementary file 18 — Additional file 18. Pearson correlations of count values obtained for three biological replicates for all whitefly infestation and hormone treatments. (a) Correlations for SA and JA treatments (0, 0.5, 1, 2, 4, 8, 12, 24 h). (b) Correlations for whitefly infestations (0, 1, 7, 14, and 22 d) for COL2246, COL1468, 60444, and TME3. [file 12864_2019_6443_MOESM18_ESM.pdf]
