# Supplementary material for: Genome-wide analyses of cassava Pathogenesis-related (PR) gene families reveal core transcriptome responses to whitefly infestation, salicylic acid and jasmonic acid
Source: BMC Genomics. 2020 Jan 29;21:93. doi: 10.1186/s12864-019-6443-1 (PMC6990599; doi:10.1186/s12864-019-6443-1)
Supplement: Supplementary file 10 — Additional file 10 PR-1, PR-2 and PR-3 family member phylogenies and consolidated gene expression heatmaps are displayed. Genes within a clade are designated by a letter and color bars in the circular phylogenetic trees and heatmaps. Information about physical clustering and cassava-specific expansions are provided beside the heatmaps, which provide gene expression changes during biotic stresses or hormone treatments (SA and JA) and in shoots and storage roots. Recent PR family expansions are shown in red in the circular trees and expansion column; other genes (light grey) in the expansion column are not part of cassava-specific PR family expansions (see Methods). Genes belonging to the same physical cluster are denoted with the same color in the cluster column; genes that do not belong to a cluster are in light grey. Genes displayed as dark grey do not have an assigned chromosomal position in the cassava genome ver. 6. Figure S15. PR-1. Figure S16. PR-2. Figure S17. PR-3. [file 12864_2019_6443_MOESM10_ESM.pdf]

PR-1

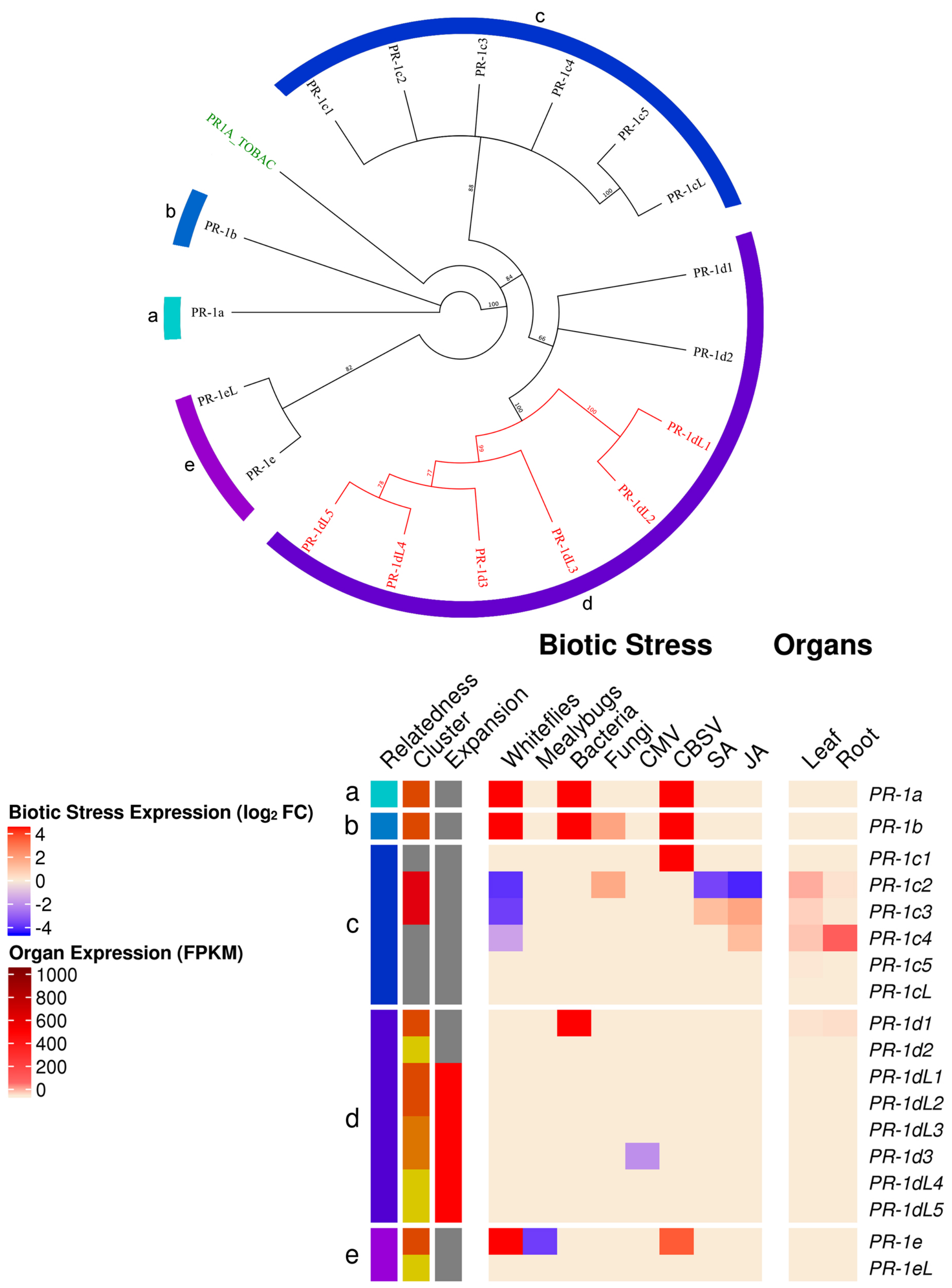

c
PR-1c1
PR-1c2
PR-1c3
PR-1c4
PR-1c5
PR-1cL
PR1A_TOBAC
b
PR-1b
a
PR-1a
PR-1d1
PR-1d2
PR-1eL
PR-1e
e
PR-1dL1
PR-1dL2
PR-1dL3
PR-1d3
PR-1dL4
PR-1dL5
d
Biotic Stress
Organs
Relatedness
Cluster
Expansion
Whiteflies
Mealybugs
Bacteria
Fungi
CMV
CBSV
SA
JA
Leaf
Root
Biotic Stress Expression (log2 FC)
4
2
0
-2
-4
Organ Expression (FPKM)
1000
800
600
400
200
0
PR-1a
PR-1b
PR-1c1
PR-1c2
PR-1c3
PR-1c4
PR-1c5
PR-1cL
PR-1d1
PR-1d2
PR-1dL1
PR-1dL2
PR-1dL3
PR-1d3
PR-1dL4
PR-1dL5
PR-1e
PR-1eL

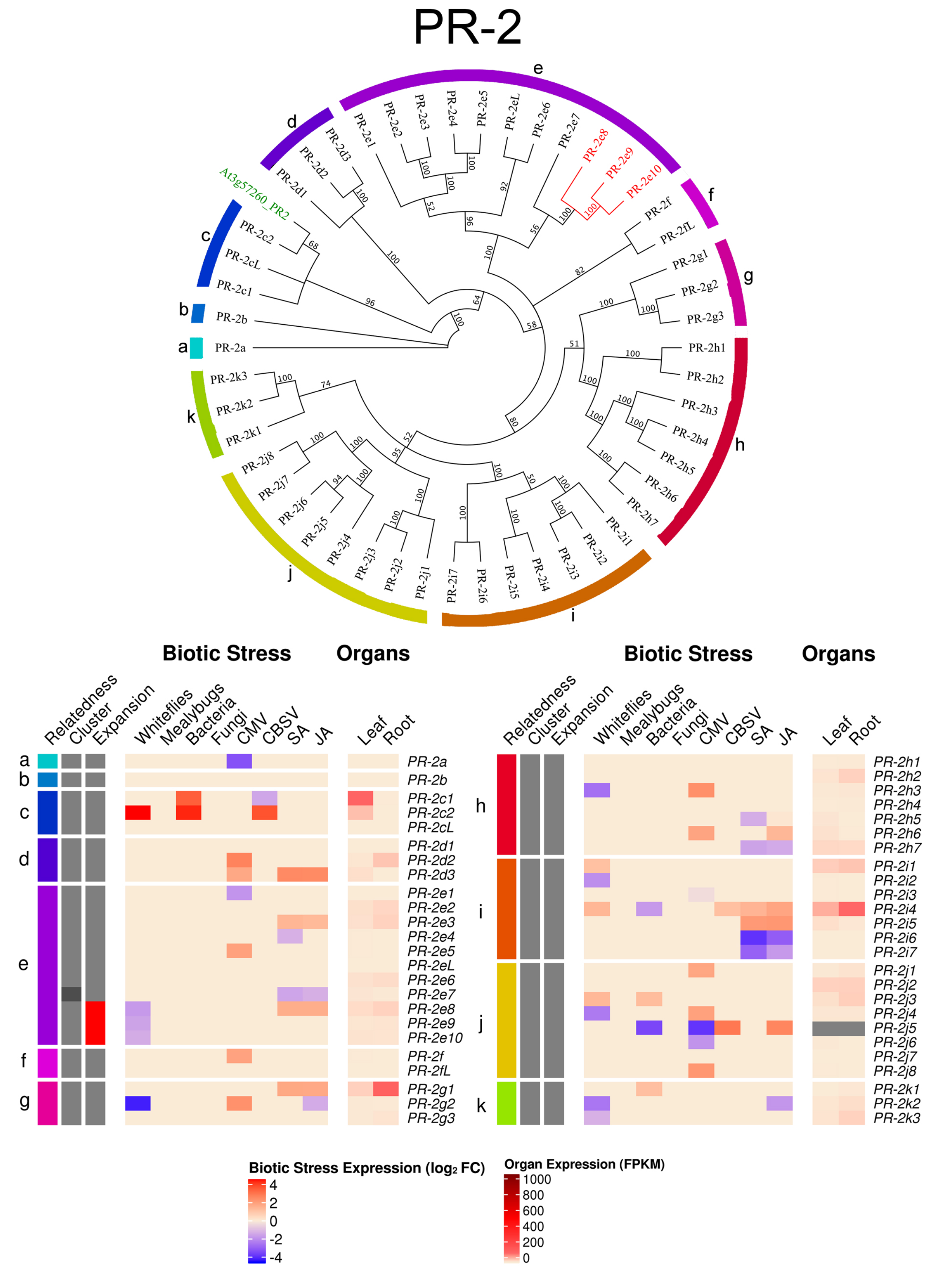

PR-2
Biotic Stress
Organs
Relatedness
Cluster
Expansion
Whiteflies
Mealybugs
Bacteria
Fungi
CMV
CBSV
SA
JA
Leaf
Root
Biotic Stress Expression (log2 FC)
Organ Expression (FPKM)

PR-3

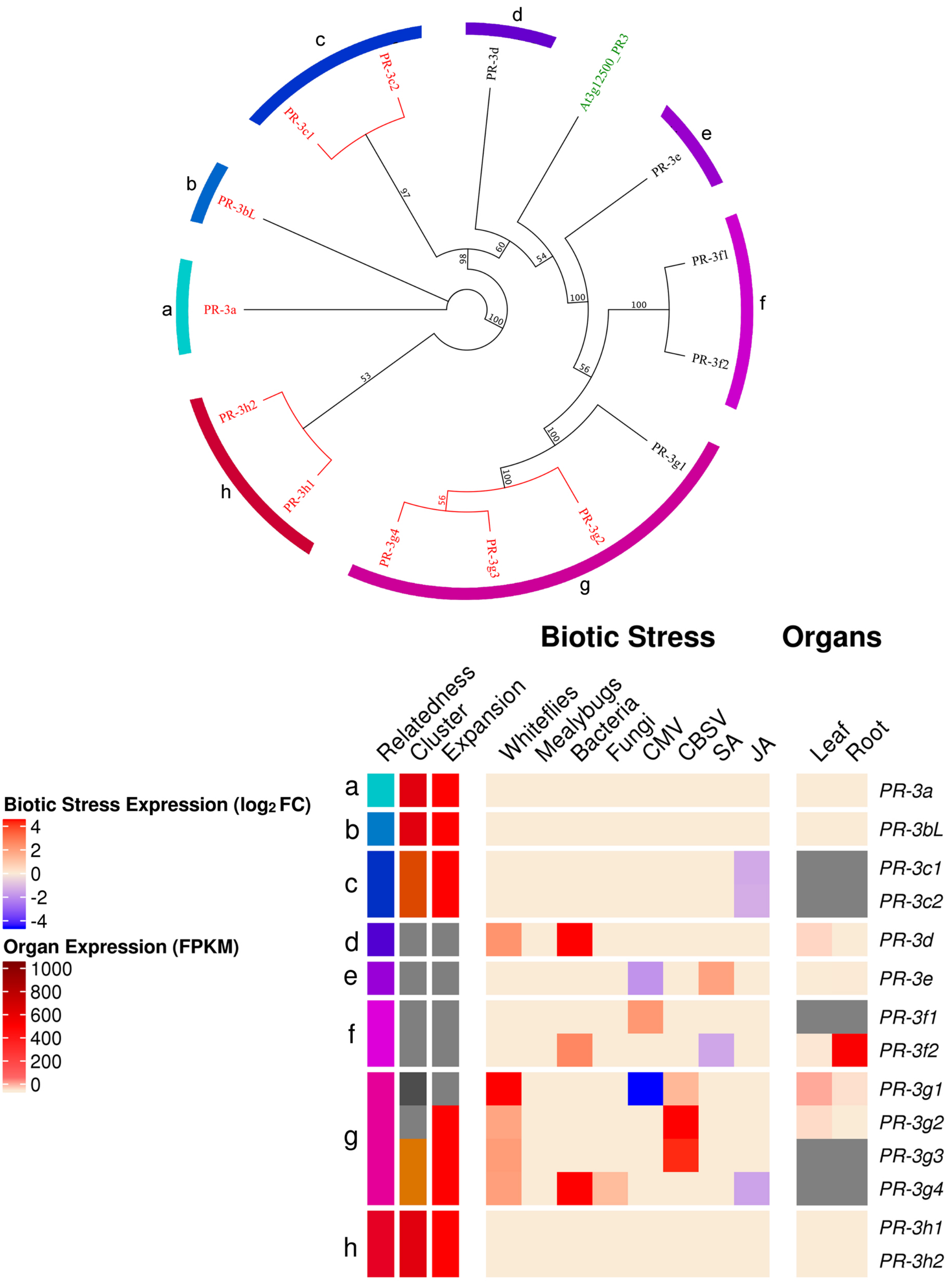

a
b
c
d
e
f
g
h
PR-3a
PR-3bL
PR-3c1
PR-3c2
PR-3d
At3g12500_PR3
PR-3e
PR-3f1
PR-3f2
PR-3g1
PR-3g2
PR-3g3
PR-3g4
PR-3h1
PR-3h2
97
98
60
54
100
100
100
56
100
100
56
53
Biotic Stress
Organs
Relatedness
Cluster
Expansion
Whiteflies
Mealybugs
Bacteria
Fungi
CMV
CBSV
SA
JA
Leaf
Root
Biotic Stress Expression (log2 FC)
4
2
0
-2
-4
Organ Expression (FPKM)
1000
800
600
400
200
0
