# Supplementary material for: Genome-wide analyses of cassava Pathogenesis-related (PR) gene families reveal core transcriptome responses to whitefly infestation, salicylic acid and jasmonic acid
Source: BMC Genomics. 2020 Jan 29;21:93. doi: 10.1186/s12864-019-6443-1 (PMC6990599; doi:10.1186/s12864-019-6443-1)
Supplement: Supplementary file 11 — Additional file 11 PR-4, PR-5 and PR-6 family member phylogenies and consolidated gene expression heatmaps are displayed. The PR-6 family phylogenetic tree is not displayed due to its small size. Figure S18. PR-4. Figure S19. PR-5. Figure S20. PR-6. [file 12864_2019_6443_MOESM11_ESM.pdf]

PR-4

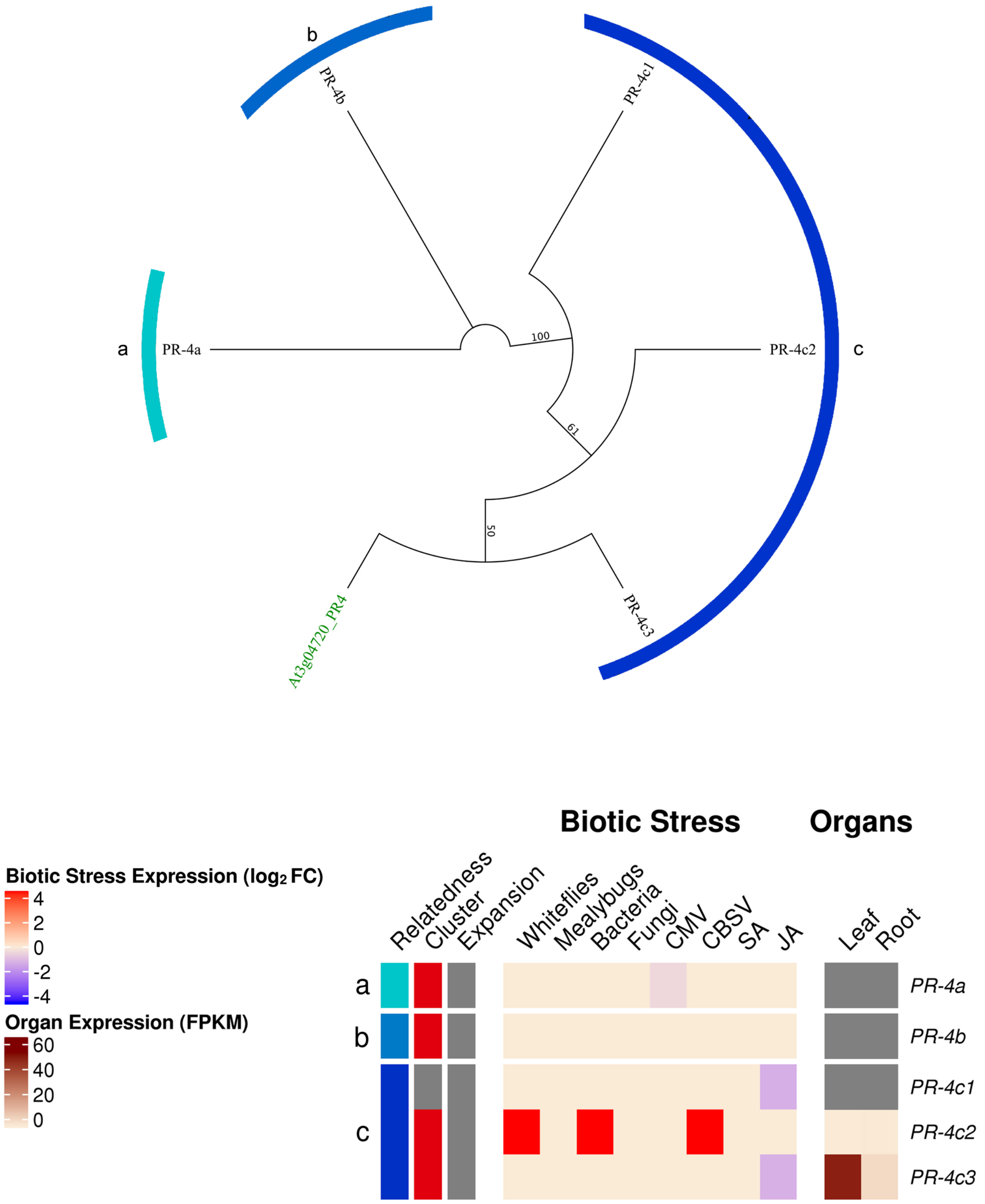

a
b
c
PR-4a
PR-4b
PR-4c1
PR-4c2
PR-4c3
At3g04720_PR4
100
61
50
Biotic Stress Expression (log2 FC)
4
2
0
-2
-4
Organ Expression (FPKM)
60
40
20
0
Biotic Stress
Organs
Relatedness
Cluster
Expansion
Whiteflies
Mealybugs
Bacteria
Fungi
CMV
CBSV
SA
JA
Leaf
Root

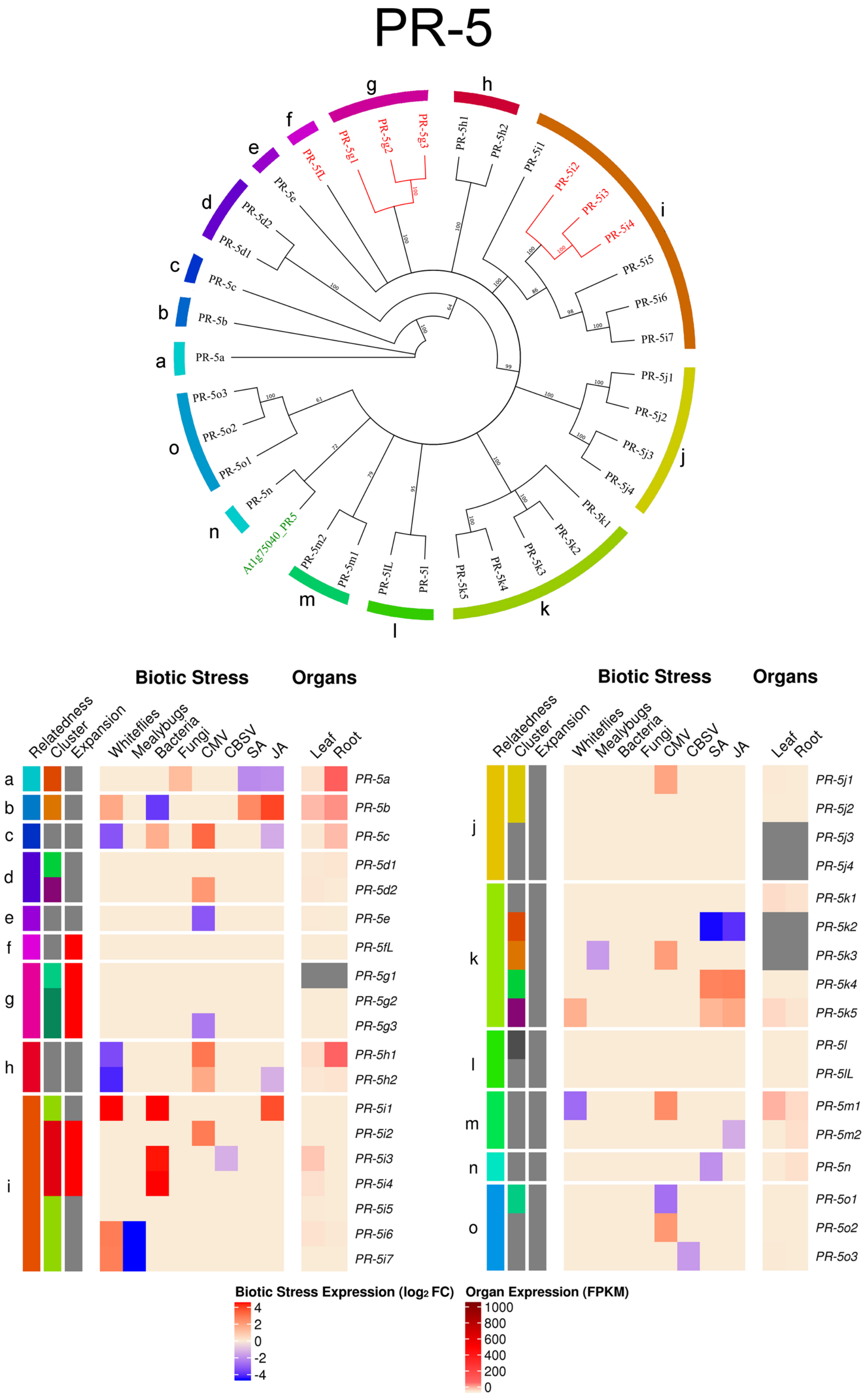
PR-5
a
b
c
d
e
f
g
h
i
j
k
l
m
n
o
PR-5a
PR-5b
PR-5c
PR-5d1
PR-5d2
PR-5e
PR-5fL
PR-5g1
PR-5g2
PR-5g3
PR-5h1
PR-5h2
PR-5i1
PR-5i2
PR-5i3
PR-5i4
PR-5i5
PR-5i6
PR-5i7
PR-5j1
PR-5j2
PR-5j3
PR-5j4
PR-5k1
PR-5k2
PR-5k3
PR-5k4
PR-5k5
PR-5l
PR-5lL
PR-5m1
PR-5m2
PR-5n
At1g75040_PR5
PR-5o1
PR-5o2
PR-5o3
Biotic Stress
Organs
Relatedness
Cluster
Expansion
Whiteflies
Mealybugs
Bacteria
Fungi
CMV
CBSV
SA
JA
Leaf
Root
Biotic Stress Expression (log2 FC)
4
2
0
-2
-4
Organ Expression (FPKM)
1000
800
600
400
200
0

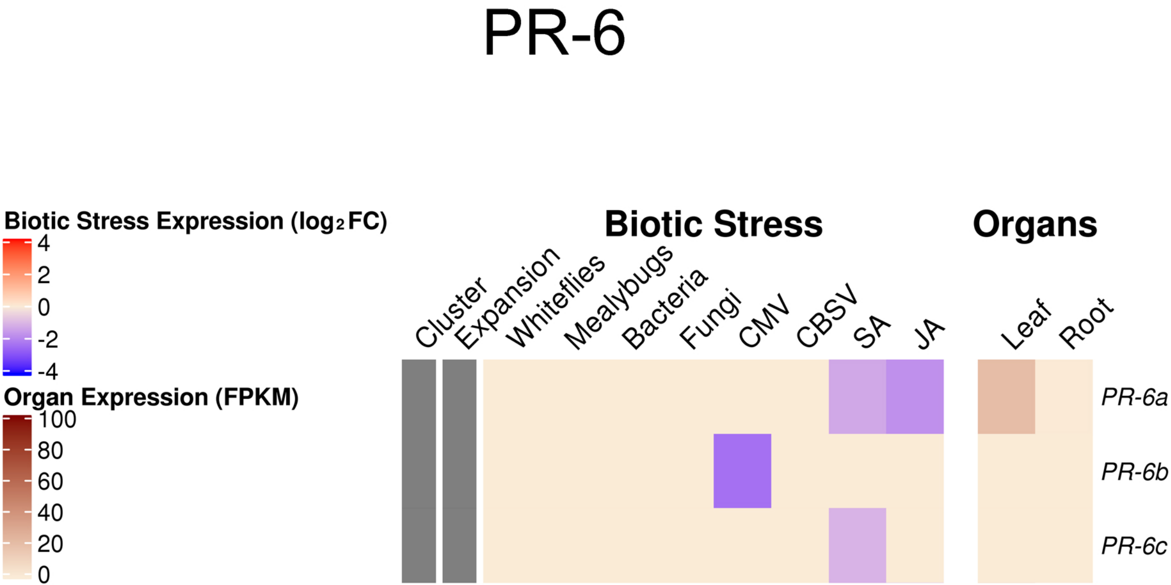

PR-6
Biotic Stress Expression (log2FC)
4
2
0
-2
-4
Organ Expression (FPKM)
100
80
60
40
20
0
Biotic Stress
Organs
Cluster
Expansion
Whiteflies
Mealybugs
Bacteria
Fungi
CMV
CBSV
SA
JA
Leaf
Root
PR-6a
PR-6b
PR-6c
