# Supplementary material for: Genome-wide analyses of cassava Pathogenesis-related (PR) gene families reveal core transcriptome responses to whitefly infestation, salicylic acid and jasmonic acid
Source: BMC Genomics. 2020 Jan 29;21:93. doi: 10.1186/s12864-019-6443-1 (PMC6990599; doi:10.1186/s12864-019-6443-1)
Supplement: Supplementary file 13 — Additional file 13 PR-9, PR-10 and PR-11 family member phylogenies and consolidated gene expression heatmaps are displayed. Figure S23. PR-9. Figure S24. PR-10. Figure S25. PR-11. [file 12864_2019_6443_MOESM13_ESM.pdf]

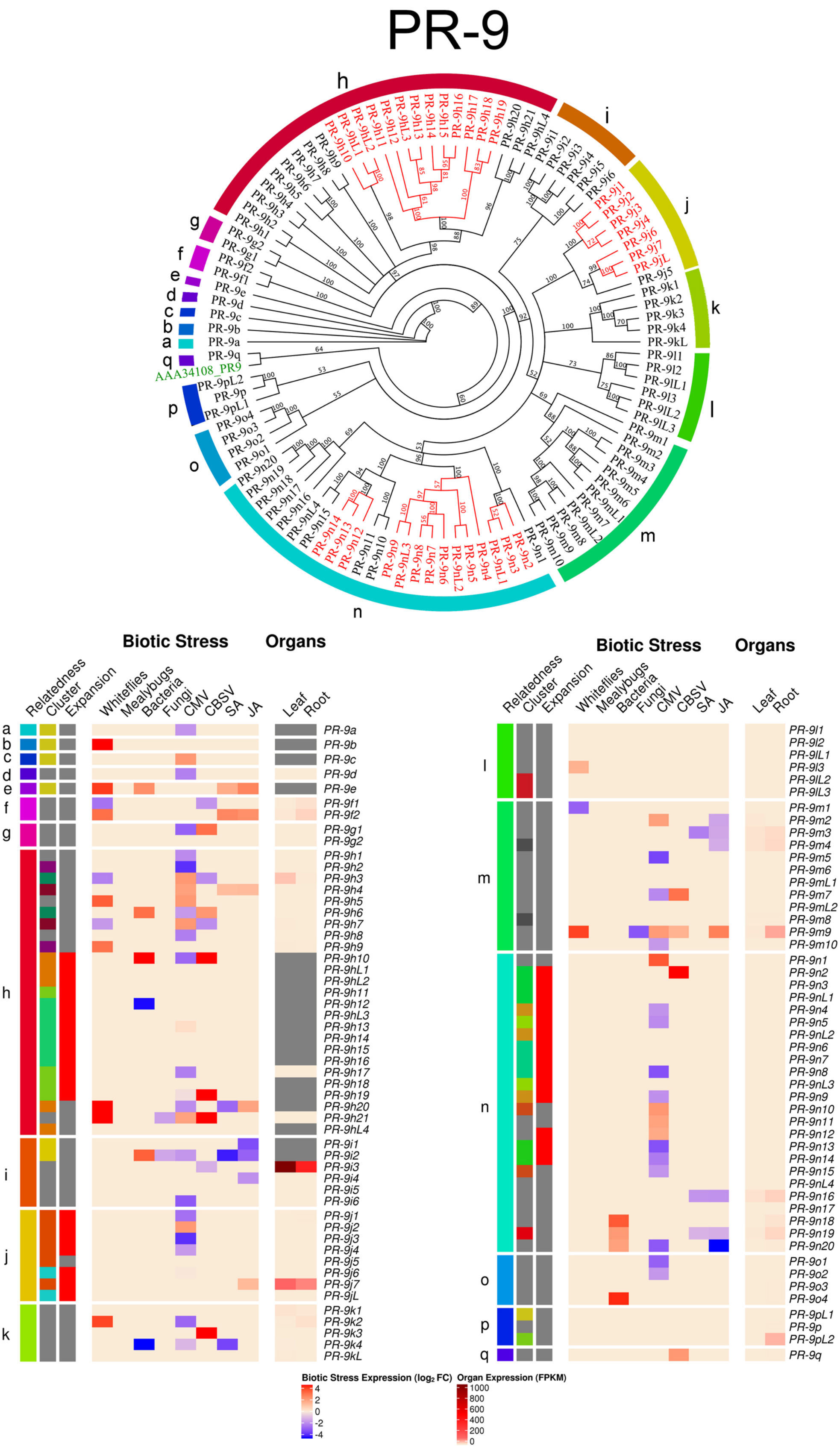

PR-9
h
i
j
k
l
m
n
o
p
q
a
b
c
d
e
f
g
AAA34108_PR9
Biotic Stress
Organs
Relatedness
Cluster
Expansion
Whiteflies
Mealybugs
Bacteria
Fungi
CMV
CBSV
SA
JA
Leaf
Root
PR-9a
PR-9b
PR-9c
PR-9d
PR-9e
PR-9f1
PR-9f2
PR-9g1
PR-9g2
PR-9h1
PR-9h2
PR-9h3
PR-9h4
PR-9h5
PR-9h6
PR-9h7
PR-9h8
PR-9h9
PR-9h10
PR-9hL1
PR-9hL2
PR-9h11
PR-9h12
PR-9hL3
PR-9h13
PR-9h14
PR-9h15
PR-9h16
PR-9h17
PR-9h18
PR-9h19
PR-9h20
PR-9h21
PR-9hL4
PR-9i1
PR-9i2
PR-9i3
PR-9i4
PR-9i5
PR-9i6
PR-9j1
PR-9j2
PR-9j3
PR-9j4
PR-9j5
PR-9j6
PR-9j7
PR-9jL
PR-9k1
PR-9k2
PR-9k3
PR-9k4
PR-9kL
PR-9l1
PR-9l2
PR-9lL1
PR-9l3
PR-9lL2
PR-9lL3
PR-9m1
PR-9m2
PR-9m3
PR-9m4
PR-9m5
PR-9m6
PR-9mL1
PR-9m7
PR-9mL2
PR-9m8
PR-9m9
PR-9m10
PR-9n1
PR-9n2
PR-9n3
PR-9nL1
PR-9n4
PR-9n5
PR-9nL2
PR-9n6
PR-9n7
PR-9n8
PR-9nL3
PR-9n9
PR-9n10
PR-9n11
PR-9n12
PR-9n13
PR-9n14
PR-9n15
PR-9nL4
PR-9n16
PR-9n17
PR-9n18
PR-9n19
PR-9n20
PR-9o1
PR-9o2
PR-9o3
PR-9o4
PR-9pL1
PR-9p
PR-9pL2
PR-9q
Biotic Stress Expression (log2 FC)
4
2
0
-2
-4
Organ Expression (FPKM)
1000
800
600
400
200
0

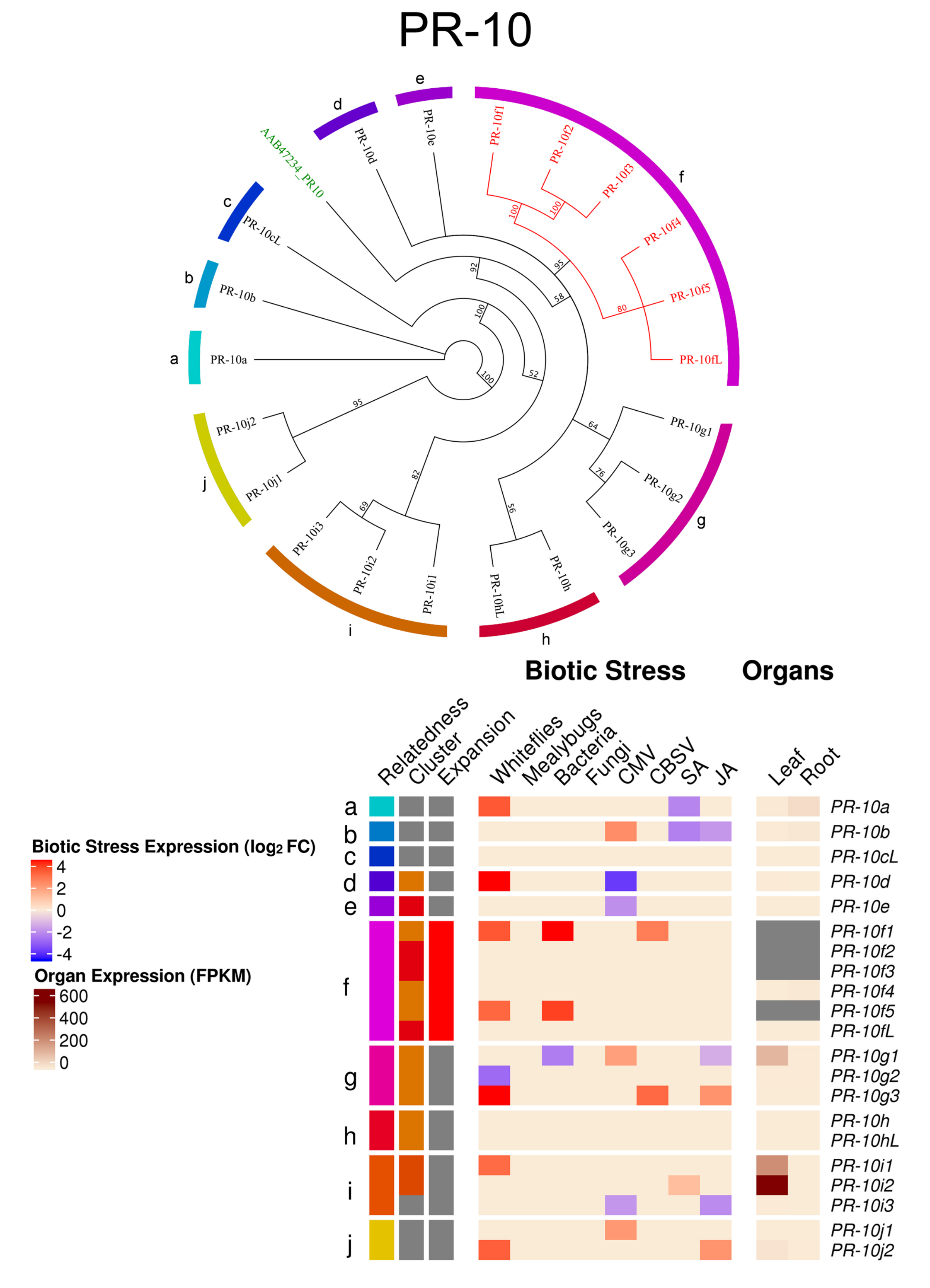

PR-10
AAB47234_PR10
PR-10a
PR-10b
PR-10cL
PR-10d
PR-10e
PR-10f1
PR-10f2
PR-10f3
PR-10f4
PR-10f5
PR-10fL
PR-10g1
PR-10g2
PR-10g3
PR-10h
PR-10hL
PR-10i1
PR-10i2
PR-10i3
PR-10j1
PR-10j2
Biotic Stress
Organs
Relatedness
Cluster
Expansion
Whiteflies
Mealybugs
Bacteria
Fungi
CMV
CBSV
SA
JA
Leaf
Root
Biotic Stress Expression (log2 FC)
4
2
0
-2
-4
Organ Expression (FPKM)
600
400
200
0

PR-11

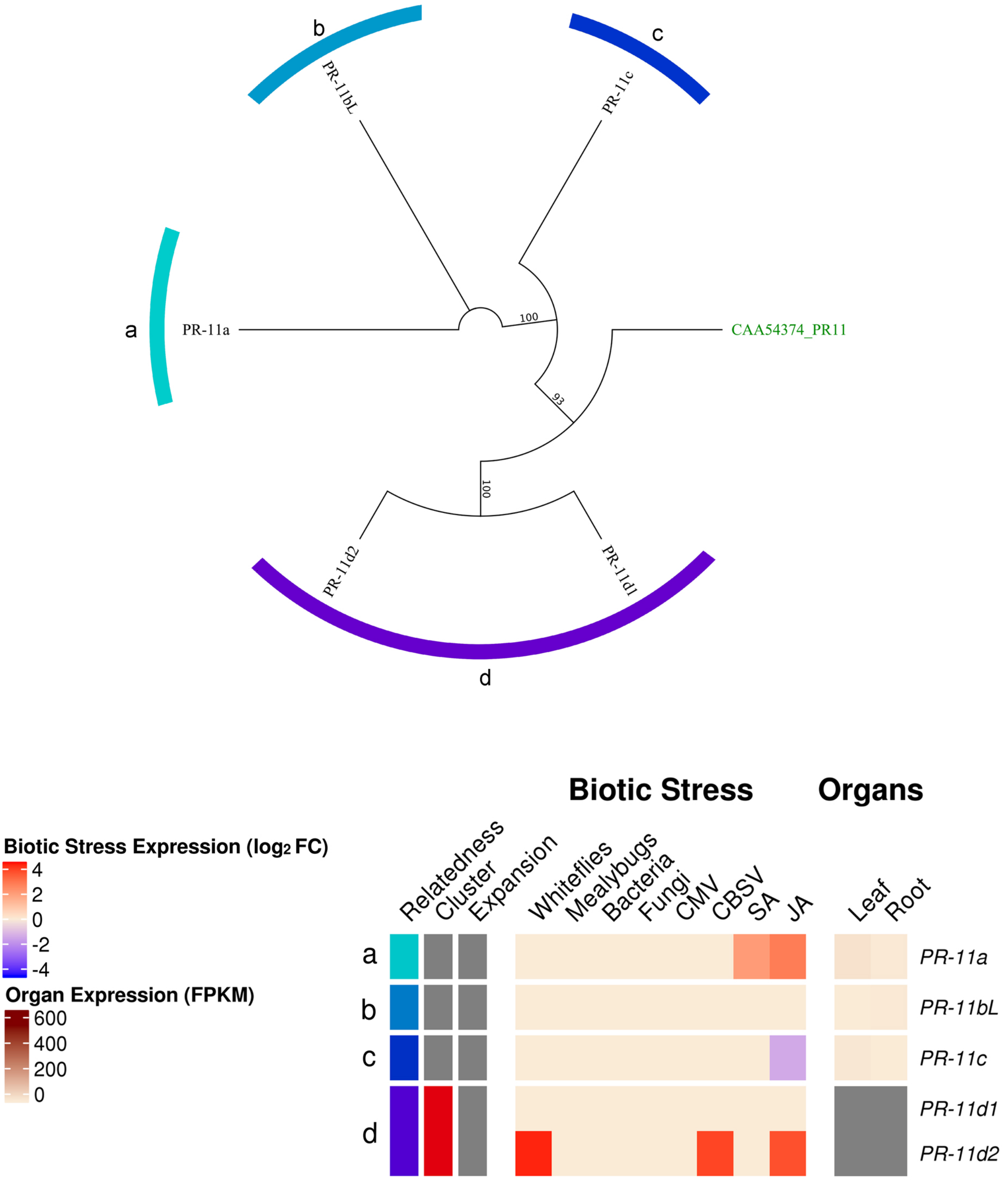

b
c
a
d
PR-11bL
PR-11c
PR-11a
CAA54374_PR11
PR-11d2
PR-11d1
100
93
100
Biotic Stress
Organs
Biotic Stress Expression (log2 FC)
4
2
0
-2
-4
Organ Expression (FPKM)
600
400
200
0
Relatedness
Cluster
Expansion
Whiteflies
Mealybugs
Bacteria
Fungi
CMV
CBSV
SA
JA
Leaf
Root
PR-11a
PR-11bL
PR-11c
PR-11d1
PR-11d2
