# Supplementary material for: Genome-wide analyses of cassava Pathogenesis-related (PR) gene families reveal core transcriptome responses to whitefly infestation, salicylic acid and jasmonic acid
Source: BMC Genomics. 2020 Jan 29;21:93. doi: 10.1186/s12864-019-6443-1 (PMC6990599; doi:10.1186/s12864-019-6443-1)
Supplement: Supplementary file 14 — Additional file 14 PR-14, PR-15/16 and PR-17 family member phylogenies and consolidated gene expression heatmaps are displayed. Figure S26. PR-14. Figure S27. PR-15/16. Figure S28. PR-17. [file 12864_2019_6443_MOESM14_ESM.pdf]

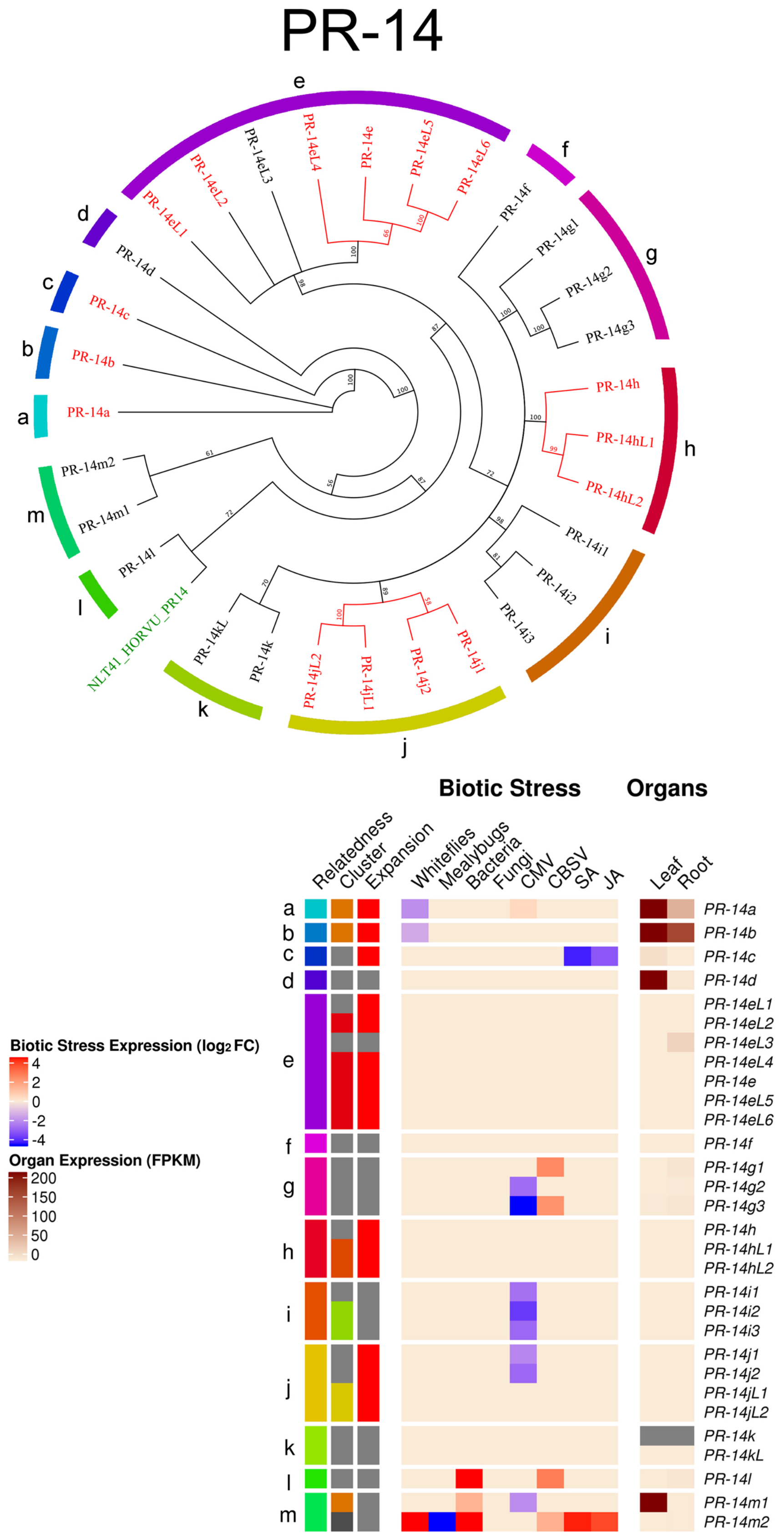

PR-14
e
f
g
h
i
j
k
l
m
a
b
c
d
PR-14eL1
PR-14eL2
PR-14eL3
PR-14eL4
PR-14e
PR-14eL5
PR-14eL6
PR-14f
PR-14g1
PR-14g2
PR-14g3
PR-14h
PR-14hL1
PR-14hL2
PR-14i1
PR-14i2
PR-14i3
PR-14j1
PR-14j2
PR-14jL1
PR-14jL2
PR-14k
PR-14kL
NLT41_HORVU_PR14
PR-14l
PR-14m1
PR-14m2
PR-14a
PR-14b
PR-14c
PR-14d
Biotic Stress
Organs
Relatedness
Cluster
Expansion
Whiteflies
Mealybugs
Bacteria
Fungi
CMV
CBSV
SA
JA
Leaf
Root
Biotic Stress Expression (log2 FC)
4
2
0
-2
-4
Organ Expression (FPKM)
200
150
100
50
0

PR-15/16

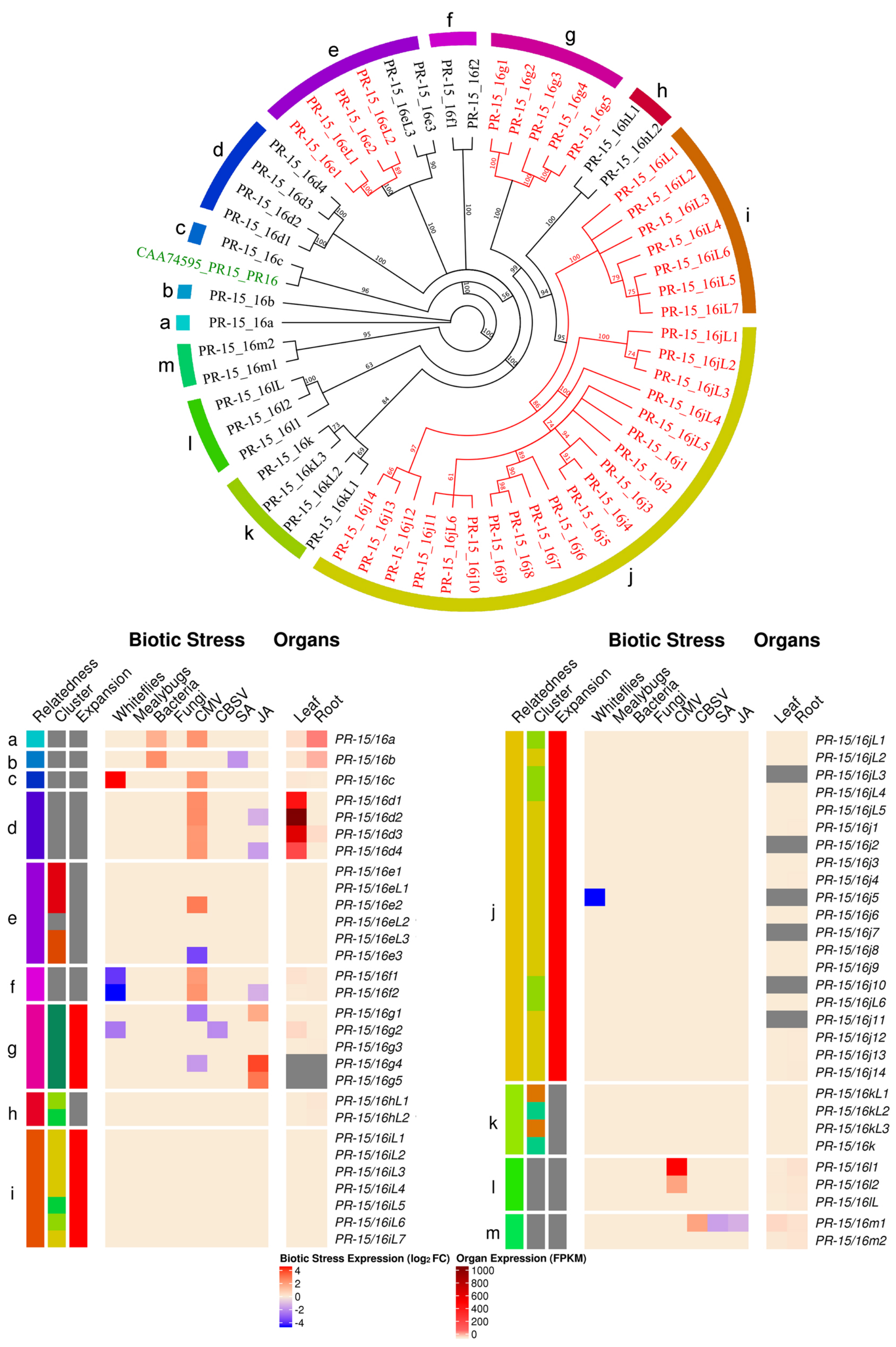

Biotic Stress
Organs
Relatedness
Cluster
Expansion
Whiteflies
Mealybugs
Bacteria
Fungi
CMV
CBSV
SA
JA
Leaf
Root
Biotic Stress Expression (log2 FC)
Organ Expression (FPKM)

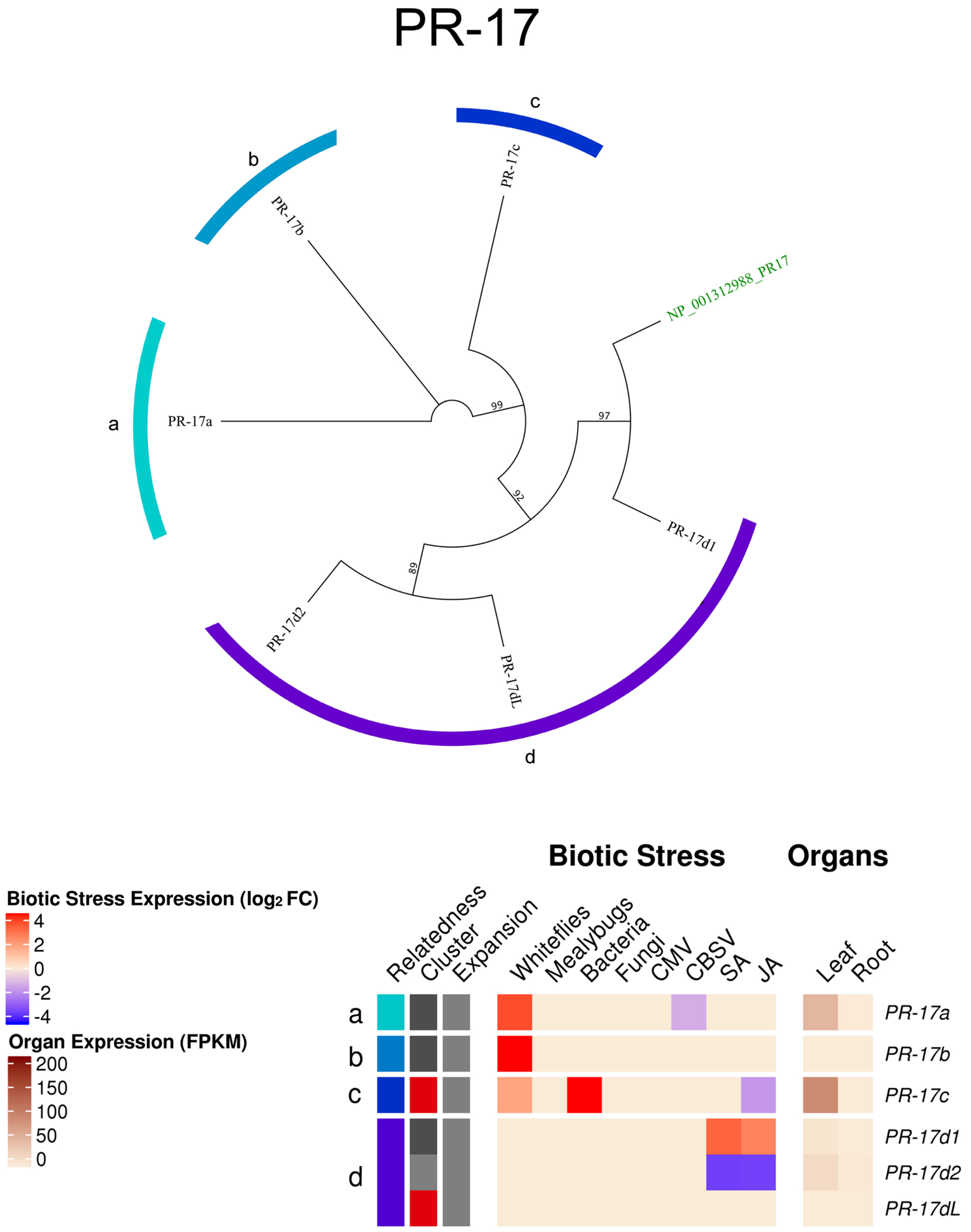

PR-17
a
b
c
d
PR-17a
PR-17b
PR-17c
NP_001312988_PR17
PR-17d1
PR-17d2
PR-17dL
99
97
92
89
Biotic Stress
Organs
Biotic Stress Expression (log2 FC)
4
2
0
-2
-4
Organ Expression (FPKM)
200
150
100
50
0
Relatedness
Cluster
Expansion
Whiteflies
Mealybugs
Bacteria
Fungi
CMV
CBSV
SA
JA
Leaf
Root
a
b
c
d
PR-17a
PR-17b
PR-17c
PR-17d1
PR-17d2
PR-17dL
